# Mapping of the Lipid-Binding Regions of the Antifungal Protein NFAP2 by Exploiting Model Membranes

**DOI:** 10.1021/acs.jcim.4c00229

**Published:** 2024-08-16

**Authors:** Olivér Pavela, Tünde Juhász, Liliána Tóth, András Czajlik, Gyula Batta, László Galgóczy, Tamás Beke-Somfai

**Affiliations:** † Institute of Materials and Environmental Chemistry, HUN-REN Research Centre for Natural Sciences, Magyar tudósok körútja 2, Budapest, H-1117, Hungary; ‡ Hevesy György PhD School of Chemistry, Eötvös Loránd University, Budapest, Pázmány Péter sétány 1/A, Budapest H-1117, Hungary; § Department of Biotechnology, Faculty of Science and Informatics, University of Szeged, Közép fasor 52, Szeged H-6726, Hungary; ∥ Department of Organic Chemistry, Faculty of Science and Technology, University of Debrecen, Egyetem tér 1 Debrecen H-4032, Hungary; ⊥ Department of Biochemistry, Institute of Biochemistry and Molecular Biology, Semmelweis University, Tűzoltó u. 37-47, Budapest H-1094, Hungary; # Institute of Biochemistry, HUN-REN Biological Research Centre, Temesvári krt. 62, Szeged H-6726, Hungary

## Abstract

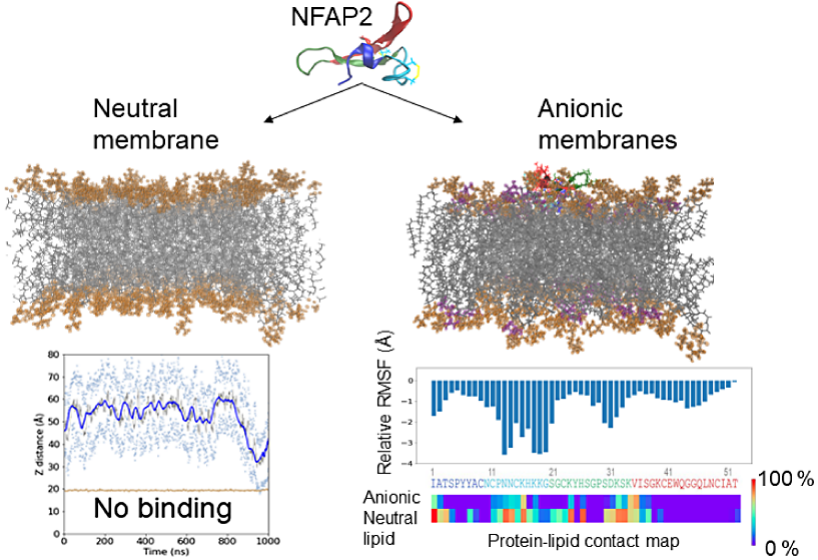

Fungal infections with high mortality rates represent an increasing health risk. The *Neosartorya (Aspergillus) fischeri* antifungal protein 2 (NFAP2) is a small, cysteine-rich, cationic protein exhibiting potent anti-*Candida* activity. As the underlying mechanism, pore formation has been demonstrated; however, molecular level details on its membrane disruption action are lacking. Herein, we addressed the lipid binding of NFAP2 using a combined computational and experimental approach to simple lipid compositions with various surface charge properties. Simulation results revealed binding preferences for negatively charged model membranes, where selectivity is mediated by anionic lipid components enriched at the protein binding site but also assisted by zwitterionic lipid species. Several potential binding routes initiated by various anchoring contacts were observed, which resulted in one main binding mode and a few variants, with NFAP2 residing on the membrane surface. Region ^10^NCPNNCKHKKG^20^ of the flexible N-terminal part of the protein showed potency to insert into the lipid bilayer, where the disulfide bond-stabilized short motif ^11^CPNNC^15^ could play a key role. In addition, several areas, including the beginning of the N-terminal (residues 1–8), played roles in facilitating initial membrane contacts. Besides, individual roles of residues such as Lys24, Lys32, Lys34, and Trp42 were also revealed by the simulations. Combined data demonstrated that the solution conformation was not perturbed markedly upon membrane interaction, and the folded part of the protein also contributed to stabilizing the bound state. Data also highlighted that the binding of NFAP2 to lipid vesicles is sensitively affected by environmental factors such as ionic strength. Electrostatic interactions driven by anionic lipids were found pivotal, explaining the reduced membrane activity observed under high salt conditions. Experimental data supported the lipid-selective binding mechanisms and pointed to salt-dependent effects, particularly to protein-assisted vesicle aggregation at low ionic strength. Our findings can contribute to the development of NFAP2-based anti-*Candida* agents and studies aiming at future medical use of peptide-based natural antifungal compounds.

## 1 Introduction

Fungal infections with high mortality represent an increasing health threat in recent decades as a consequence of the lack of effective antifungal drugs and the spread of drug-resistant strains. Among these, candidiasis caused by *Candida* species is particularly dangerous because of high infection and mortality rates observed in hospitals, linked mainly to their ability to form biofilms.^1−3^

In the fight against fungal pathogens, natural antimicrobial peptides and proteins (AMPs) represent a promising set of antifungal compounds. AMPs can show broad-spectrum activity against diverse microorganisms; many of them can be effective on bacteria and fungi.^4^ Recently, a class of short, cysteine-rich, natural antifungal proteins (AFPs) of filamentous fungi origin has been demonstrated for their potent antifungal activity.^5^ Among these, the *Neosartorya (Aspergillus) fischeri* antifungal protein 2 (NFAP2) holds high therapeutic potential as it can inhibit the growth of various human pathogenic *Candida* species, acting effectively on biofilms resistant to conventional small molecule drugs.^6,7^ Furthermore, it showed synergistic effects on planktonic and sessile cells when administered in combination with first-line anti-*Candida* drugs.^6,8^

However, to realize the potential of NFAP2 as an antifungal drug, its mechanism of action needs to be better understood at the molecular level. In this respect, AFPs, like AMPs in general, can exert various mechanisms to kill the target pathogens.^9^ Note that while they can have intracellular targets, they most often attack the cell membrane. Particularly, NFAP2 induced pore formation and lysis on planktonic *Candida* cells.^7,8^ Functional mapping of the 52 aa protein identified fragment 13–23 as the potentially active antifungal segment.^8^ According to the recent NMR structure, which aided in solving the disulfide bonding pattern of the protein, this region is partially restrained by a disulfide bridge between Cys11 and Cys15.^10^

Addressing structural and mechanistic details of the membrane interaction of NFAP2, herein we used computational and experimental methods, a combination used beneficially on several AMP–lipid interaction systems previously.^11−15^ Specifically, based on the above preliminary structure,^10^ further refinements have been made here toward a detailed NMR structure. This was then used for all-atom molecular dynamics (MD) simulations, which were accompanied by lipid binding assays exploiting spectroscopic methods. Characterization was performed on several model membranes with lipid compositions differing in the headgroup charge distribution. As NFAP2 exhibited significantly higher activity on *Candida* cells in low salt medium,^8^ environmental factors potentially affecting binding determinants were also probed upon applying conditions of various ionic strength. Results obtained here revealed details on lipid selectivity, preferred binding modes, and key interaction segments of NFAP2, contributing to a better understanding of its membrane disrupting activity.

## 2 Methods

### 2.1 Molecular Dynamics Simulations

For the simulations, the solution NMR structure of NFAP2 was used. The preliminary NMR structure reported previously^10^ was refined, as detailed in the Methods section of the Supporting Information. Parameters/coordinates of the refined structure were deposited (PDB ID 8RP9, BMRB ID 34891). The protonation states of the ionizable side chains, i.e., LYS, ASP, GLU, and HIS, were assigned by the GROMACS “gmx pdb2gmx” command, selecting the forms of the residues that are relevant at around pH 7, the physiological pH range.

All simulations were carried out by the GROMACS 2019.4 software,^16^ applying the CHARMM36m force field.^17^ The three membrane models were generated using the CHARMM-GUI website,^18^ 100% phosphatidylcholine (PC), 80/20 (*n*/*n*) PC/phosphatidylglycerol (PG), and 80/20 (*n*/*n*) PC/phosphatidylserine (PS). The bilayers consisted of 2 × 128 lipids with CHARMM-modified TIP3P water molecules and 150 mM Na^+^ and Cl^–^ ions. The membrane models were energy minimized, equilibrated, and run for 1 μs. Details on the simulations, including parameters such as long-range interaction algorithms, are given in Table S1. Subsequently, water and ions were removed, and the peptide was inserted above the bilayers. The starting distance between the center of mass (COM) of the peptide and the COM of the lipid headgroups on the peptide-facing bilayer leaflet, was always set to at least 20 Å. The systems were resolvated, and ions were added to each bilayer, either counterions only to neutralize the system or additional 150 mM Na^+^ and Cl^–^, resulting in low salt and high salt PC, PC/PG, and PC/PS systems, respectively. Next, energy minimization was done using 5000 steps with the steepest descent algorithm. Then the systems were heated up to 303.15 K temperature over 125 ps using the Berendsen thermostat.^19^ In the next step for the NPT ensemble equilibration, 1 bar of pressure was applied for 125 ps with a semiisotropic Berendsen barostat in addition to the Berendsen thermostat at 303.15 K, used in the previous step. Trajectories were collected from each system for 1 μs time, using a 2 fs time step; for PC/PG, PC/PS systems two additional parallel simulations were started from the same equilibrated files; thus, in total three high salt and three low salt 1 μs long simulations were performed for these systems, meanwhile for PC systems: only one high salt and one low salt 1 μs long simulations. Because the weak coupling algorithm employed in the Berendsen thermostat and barostat does result in a deviation from the canonical ensemble, production simulation runs were performed using the Nose–Hoover thermostat^20^ and the Parrinello–Rahman barostat algorithm.^21^ Electrostatics were treated by the Particle mesh Ewald (PME) method.^22^ The LINCS algorithm^23^ was used to constrain bonds between hydrogens and their corresponding heavy atoms. Periodic boundary conditions were applied in all directions.

Additionally, simulations of a system containing only the NFAP2 peptide without lipid bilayers were also executed using the settings described above. This consisted of two simulations, one NFAP2 system with 150 mM Na^+^ and Cl^–^ ions, and one only neutralized, both having a 1 μs long production run time.

Analysis of the trajectories was performed using the MDAnalysis Python package.^24,25^ The analysis programs were implemented in Jupyter notebooks.

The distance between the protein and the surface of the bilayer was defined as the absolute value of the distance between the z-coordinate of the center of mass (COM) of the peptide atoms and the average z-coordinate of the phosphorus atoms of the leaflet that is closer to the peptide. Contacts between an amino acid residue and a lipid residue were defined by having at least one pair of heavy atoms (non-hydrogen atoms) being closer than 4 Å to each other from those two particular residues.

Images illustrating simulation results were produced using Visual Molecular Dynamics (VMD).^26^

### 2.2 Peptide and Lipid Solutions

Recombinant NFAP2 was produced according to Tóth et al.^7^ and additionally purified with reversed-phase high-performance liquid chromatography.^6^ NFAP2 solutions were prepared dissolving lyophilized NFAP2 in either ultrapure water (Milli-Q) or in PBS (10 mM phosphate, 140 mM NaCl, 3 mM KCl, pH 7.4) at 1 mM and stored frozen at −18 °C.

High-purity synthetic 1,2-dioleoyl-*sn*-glycero-3-phosphocholine (DOPC), 1,2-dioleoyl-*sn*-glycero-3-[phospho-*rac*-(1-glycerol)], sodium salt (DOPG), and 1,2-dioleoyl-*sn*-glycero-3-phospho-l-serine (sodium salt) (DOPS) were purchased from Sigma-Aldrich. Large unilamellar vesicles of 100 nm were prepared in PBS at 10 mg/mL using the lipid film hydration method and extrusion.^27^

### 2.3 Lipid Overlay Assay

Lipid binding was probed using PIP Strips Membranes (Thermo Fisher Scientific, Waltham, MA, USA). Strips were blocked in TBST+B buffer (10 mM Tris-HCl, pH 8.0, 150 mM NaCl, 0.1% Tween 20, 3% bovine serum albumin) for 1 h at room temperature, then NFAP2 was introduced at 5 μg/mL in TBST+B for 4 h at room temperature. The membrane was washed with TBST+B three times for 10 min and incubated with affinity purified NFAP2 antiserum 1:1000 in TBST+B (1.13 mg/mL; produced in rabbit (Davids Biotechnologie GmbH, Regensburg, Germany) at 4 °C overnight. The membrane was washed with TBST+B three times for 10 min at room temperature and incubated with Anti-Rabbit IgG (whole molecule)–Alkaline Phosphatase antibody produced in goat (Sigma-Aldrich, St. Louis, MO, USA) 1:10 000 in TBST+B for 1 h at room temperature. The membrane was washed again with TBST+B three times for 10 min and equilibrated with substrate buffer (100 mM Tris-HCl pH 8.3, 150 mM NaCl, 1 mM MgCl_2_) for 5 min at room temperature. Detection was performed with 1-Step NBT/BCIP solution (Thermo Fisher Scientific, Waltham, MA, USA) and stopped with dH_2_O. The membrane was gently shaken (45 rpm) in all steps. As a control, water-diluted 1 μg of NFAP2 was applied and dried on the membrane.

### 2.4 Circular Dichroism Spectroscopy

Spectra were collected in the far-UV region (195–250 nm) using a JASCO J-1500 spectropolarimeter at room temperature. NFAP2 and lipid concentrations were 25 and 635 μM, respectively, in PBS or 20× PBS. Spectra were recorded at a speed of 50 nm/min with a bandwidth of 1 nm using a cylindrical quartz cuvette of 1 mm path-length, corrected by subtracting a matching blank, and smoothed. Secondary structure prediction from CD spectra was performed using the BeStSel method^28^ available at https://bestsel.elte.hu.

### 2.5 Attenuated Total Reflectance Fourier-Transform Infrared (ATR-FTIR) Spectroscopy

ATR-FTIR spectra were acquired using a Varian 2000 FTIR Scimitar Series spectrometer with a Golden Gate single reflection diamond ATR accessory (Specac Ltd., Orpington, UK). Three microliter sample (25 μM NFAP2, 635 μM lipid, in PBS or 20× PBS) was mounted on the diamond ATR crystal and left to dry upon slow evaporation of the solvent water under ambient conditions. To collect spectra, 64 scans were coadded at a nominal resolution of 2 cm^–1^. Spectra were analyzed with the GRAMS/32 software package (Galactic Inc., USA) and the Origin 2020 software (OriginLab, Northampton, MA, US).

Recording ATR-FTIR spectra for hydrated proteins and lipids in dry film enhances sensitivity by eliminating the major contributions from the aqueous medium. Importantly, during the gentle drying process, primarily bulk water is removed, whereas the native hydration shell of the solute is highly preserved.^29^ The latter also implies that spectral variations observed for the dry film could report on the interactions formed in the solution. Nevertheless, higher local concentrations in the dry film might induce extra contacts in comparison with the diluted solutions.

### 2.6 Fluorescence Spectroscopy

Spectra were collected using a Jasco FP-8500 spectrofluorometer at 25 °C. NFAP2 and lipid concentrations were 5 and 125 μM, respectively, in PBS or 100× PBS. The tryptophan fluorophore was excited at 280 nm, and emission was recorded between 305 and 400 nm. Three spectra were averaged and corrected by subtracting a matching blank, and the emission intensity at the maximum was read.

### 2.7 Dynamic Light Scattering

Particle size distribution was measured at 20 °C in disposable cuvettes with a 1 cm path-length (UVette, Eppendorf Austria) using a W130i dynamic light scattering (DLS) device (Avid Nano Ltd., High Wycombe, UK) equipped with a diode laser (660 nm) and a photodiode detector. NFAP2 and lipid concentrations were 25 and 635 μM, respectively, in PBS or 20× PBS. The autocorrelation function was measured for 10 × 10 s. Data analysis was performed with the iSize 3.0 software supplied with the device.

## 3 Results and Discussion

### 3.1 Lipid Selection for Membrane Binding of NFAP2

**Figure 1:**
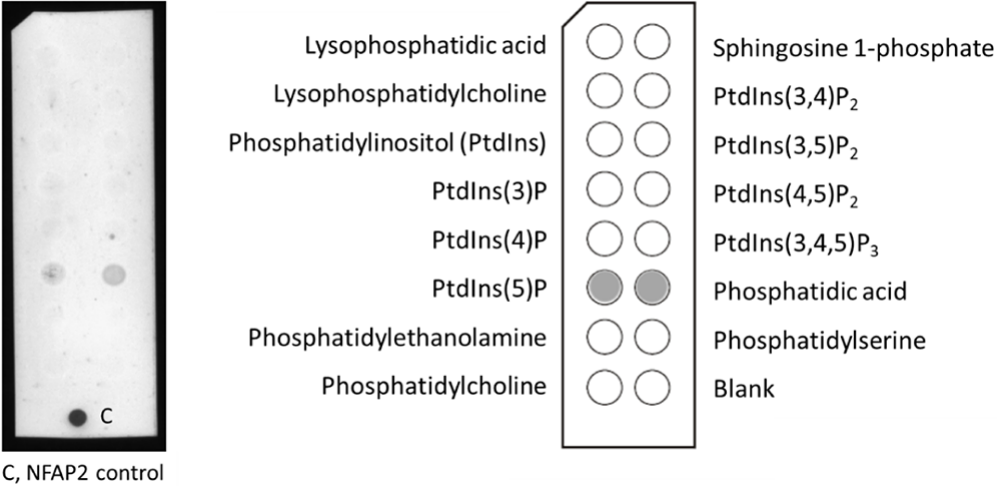
Lipid selectivity of NFAP2 obtained from lipid overlay assays. Binding to phosphatidylinositol 5-phosphate (PtdIns(5)P) and phosphatidic acid (PA) was detected. For detection control, NFAP2 was also applied.

**Figure 2:**
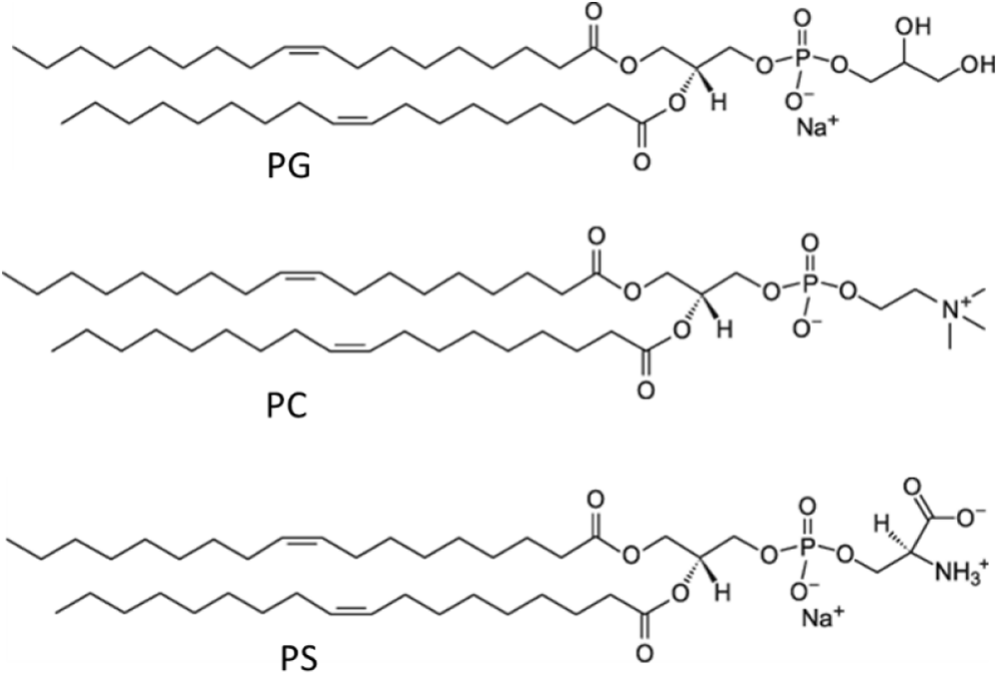
Structure of the lipids used in the study. Simulations and experiments were performed on lipid bilayers PC, PC/PG, and PC/PS composed of phosphatidylglycerol (PG), phosphatidylcholine (PC), and phosphatidylserine (PS). For details on the composition ratio, see the Methods section.

Seeking lipid components that could contribute to the membrane binding of NFAP2, a protein lipid overlay assay was utilized first. Upon testing glycerophospholipids, including phosphatidylinositols, no binding to neutral lipids such as PC, lysophosphatidylcholine (LPC), or phosphatidylethanolamine (PE) was observed, while interaction with layers composed of phosphatidylinositol 5-phosphate (PtdIns(5)P, PIP5) and phosphatidic acid (PA) could clearly be detected (Figure 1 and Figure 2). Interestingly, no interaction with higher phosphorylated phosphatidylinositols (bis- or trisphosphates) was detected either. PA has a single phosphate group in its headgroup, while in PIP5, an extra phosphate is coupled to the inositol ring that extends the basic phosphate moiety. This indicates a preference for NFAP2 for anionic lipids with exposed phosphate groups. It is to be noted that the lipid strip assay offers a useful initial qualitative insight into lipid binding preference; however, it might obtain false negative results.^30^ Accordingly, other anionic lipids presented in the strip, i.e., PS, not identified as a strong binding partner in this assay, could also show affinity for NFAP2 when introduced in the solution phase.

**Figure 3:**
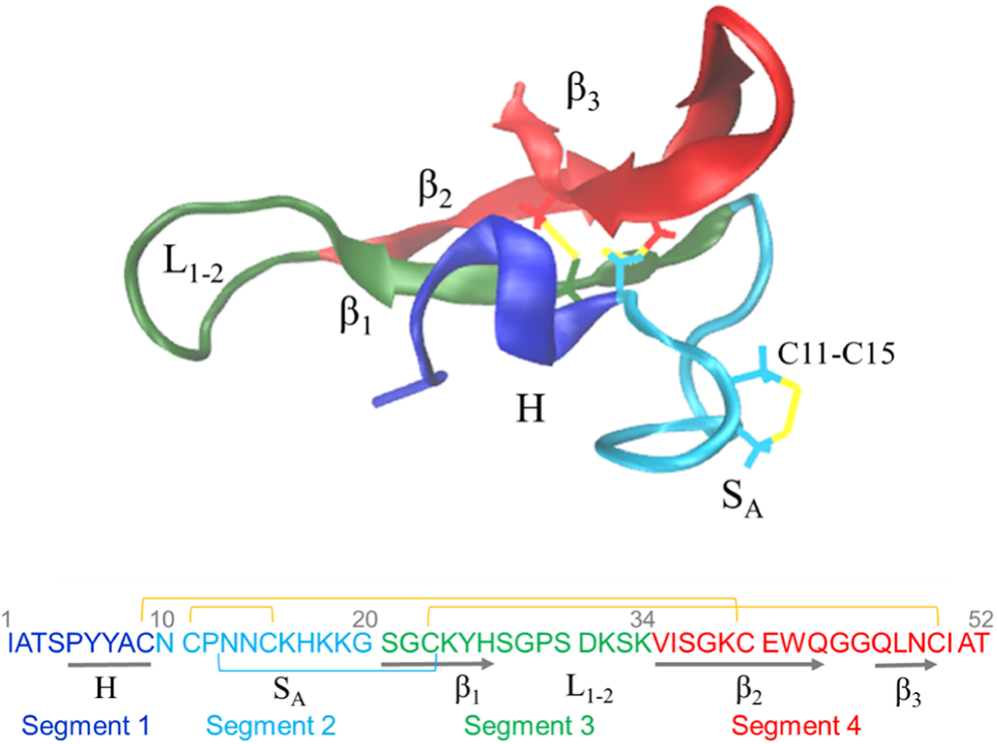
Solution structure of NFAP2. The NFAP2 sequence was divided into four segments based on structural aspects as follows: segment 1 (res 1–9, blue) contains the N-terminal part, including a short sequence motif showing helical tendency (H); segment 2 (res 10–20, cyan) is a long, flexible loop that corresponds eventually to the antifungal, active fragment (res 13–23, S_A_); segment 3 (res 21–34, green) contains strand β_1_ and a long loop (L_1–2_) connecting β_1_ and β_2_; segment 4 (res 35–52, red) contains the strands β_2_ and β_3_. The three disulfide bridges are highlighted in yellow. Note the uncommon short stretch of the C11–C15 disulfide bond located partially in the proposed active segment.

Strip hits strongly indicate an affinity of NFAP2 toward negatively charged membrane components. Note, however, that neither PA nor PI5P is a major component of plasma membranes, as they are present in a very low percentage of the total lipid content. Considering these, the lipid selectivity of NFAP2 was probed in this study using simple glycerophospholipid compositions displaying various anionic surface charge distributions while mimicking biologically relevant cell membranes. Specifically, the applied model membranes were built of lipid species mimicking the characteristic anionic features of fungal and bacterial cell membranes, in comparison to neutral mammalian cell membranes (Figure 3). Thus, for a neutral bilayer, PC with a zwitterionic headgroup was used, representing the outer leaflet of mammalian cell membranes. For a net negatively charged bilayer, PC was mixed with anionic PS or PG. PS is abundant in the inner leaflet of resting, healthy mammalian cell membranes while it is exposed in the outer leaflet of cancerous cells, potential targets of antimicrobial peptides with anticancer activity.^31,32^ PS is also present in fungal cell membranes where it might play roles in fungal virulence as demonstrated on human pathogenic fungi e.g., *Candida albicans*.^33−36^ PS has a total negative charge, with a −/+/– charge distribution along its headgroup, according to the phosphate/amine/carboxyl moieties. PG is a component of bacterial cell membranes^37,38^ and bears a single negative charge due to an anionic phosphate group.

### 3.2 Binding Determinants of the NFAP2-Lipid Interactions Revealed in Simulations

For exploring the lipid binding of NFAP2, we applied an *in silico* approach on the selected PC, PC/PG, and PC/PS bilayers. As experimental results on the anti-*Candida* effect of NFAP2 showed significantly reduced activity in high-salt medium compared to low-salt conditions,^8^ likely linked to putative electrostatic effects mediating NFAP2 action, we explored NFAP2–lipid interactions under both low-salt and high-salt conditions. For the former, only ions needed to neutralize the system were added, whereas for the latter, 150 mM NaCl was supplemented.

#### 3.2.1 The Structure of NFAP2 Used in the Simulations

For the simulations, we refined the preliminary NMR structure of NFAP2 (Figure 3).^10^ The refined structure shows a highly flexible N-terminal half, including the active fragment, followed by the folded C-terminal half built up of three β-strands. Strand β1 is connected to β2 by a long loop, L1, while β2 is connected to β3 via a short turn. Segments 13–23, identified previously as an active region in antifungal tests, reside in the nonfolded protein half. Due to the unique disulfide pattern of NFAP2, the C11–C15 disulfide bridge introduces a short stretch with restricted mobility to the flexible protein part. Moreover, residues Pro5–Cys9 show helical tendency; however, such a short region cannot be considered a stable helix.

#### 3.2.2 Lipid Selectivity: Binding Preference of NFAP2 to Anionic Membranes

**Figure 4:**
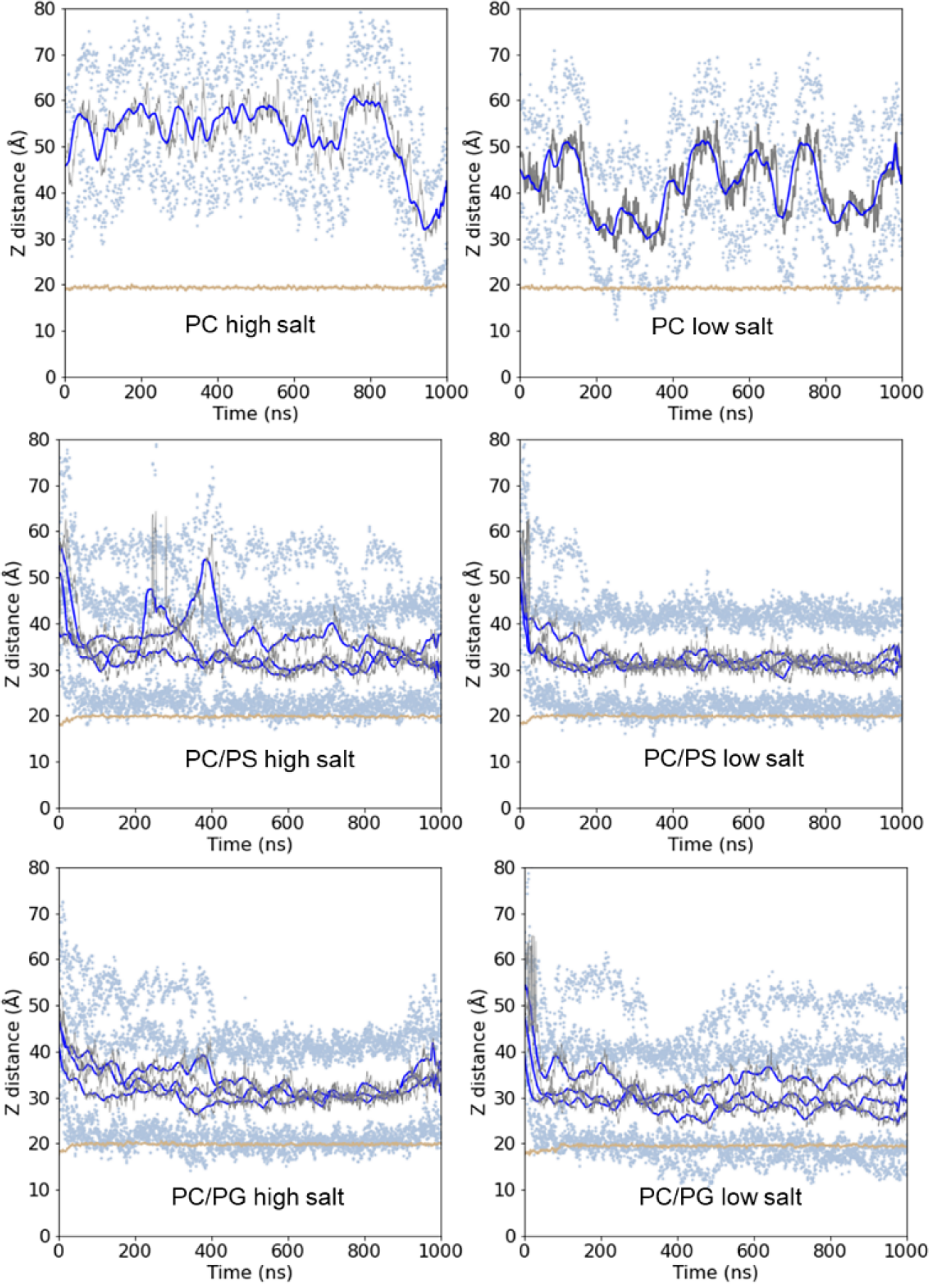
Distance of the peptide from the center of the bilayer during the simulations. Data points refer to distances from the center of the bilayer, for the peptide COM (gray) and its moving average (blue); the average distance of the 25 farthest and closest peptide atoms (light blue); the COM of the phosphorus atoms in the headgroup region of the lipid bilayer facing NFAP2 (tan). For the anionic systems, data for all three parallel simulations are shown.

First information about the lipid-binding ability of NFAP2 was obtained from the analysis of the distance of the protein from the center of the lipid bilayer during the simulations (Figure 4). With the zwitterionic PC bilayer under high-salt conditions, no strong membrane interaction of NFAP2 was observed. The protein bound to the bilayer surface only for a few nanoseconds at the end of the 1 μs simulation period, but then it quickly dissociated. Under low-salt conditions, NFAP2 binding was more pronounced, and the protein was able to contact the PC bilayer for longer but still rather short (typically 50–70 ns) time periods. In contrast, NFAP2 favored binding to the anionic PC/PG and PC/PS membranes at both low and high ionic strength, which manifested in stable association without further dissociation events. To investigate the nature of NFAP2 binding to these bilayers in detail, three parallel simulations were run and analyzed for each condition for the PC/PG and PC/PS systems.

Among the anionic systems, the smallest distance values of the center of mass (COM) of the peptide to the bilayer were obtained for PC/PG, which suggested stronger binding compared to the PC/PS systems. In the PC/PG systems, NFAP2 was partially inserted into the membrane, as indicated by lower COM values for the residues lying closest to the lipids compared to the bilayer surface (Figure 4). For the PC/PS systems, higher distance values indicated that NFAP2 was located rather on the membrane surface without significant insertion. Moreover, for PC/PS under high-salt conditions, the increased COM values suggested the dissociation of the protein from the membrane for 100–200 ns. This observation is further indicative of (i) looser binding to PC/PS compared to PC/PG, showing no dissociation events during the course of simulations once associated with the bilayer, and (ii) stronger binding at low ionic strength. In line with the latter observation, we can note that for each tested lipid bilayer pair, lower distances, likely pointing to stronger membrane affinity were, calculated under low-salt conditions.

#### 3.2.3 Contact Mapping of NFAP2 in Binding to Anionic Lipid Bilayers

**Figure 5:**
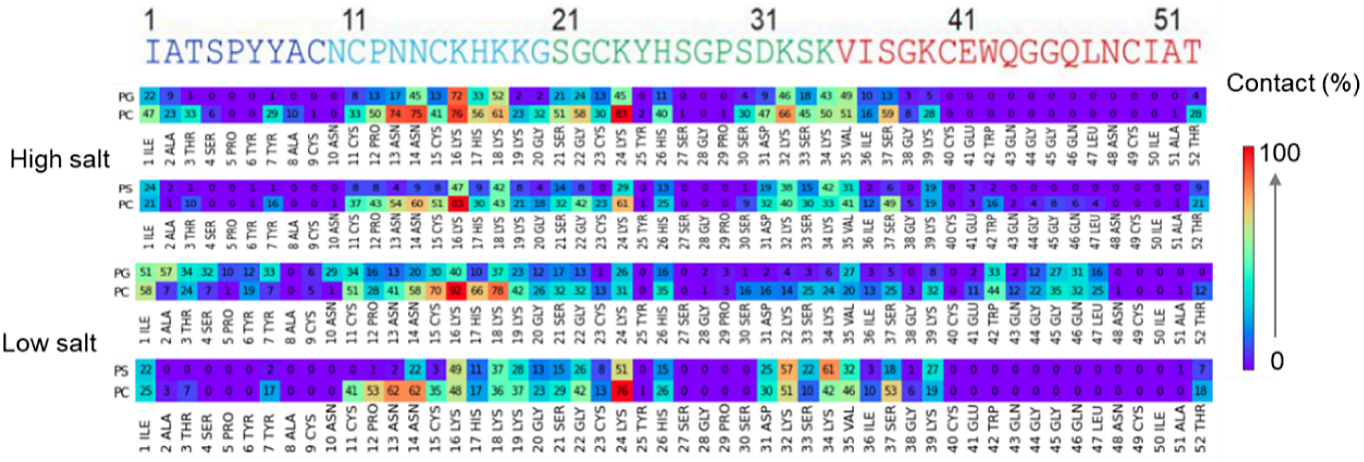
Contact analysis on NFAP2 upon binding to anionic model membranes. Values represent the percentage of frames in which contacts formed between any residue of NFAP2 and a particular lipid. Contacts were calculated for the last 500 frames, corresponding to the last 500 ns of the simulations, with values averaging from three independent simulations.

**Figure 6:**
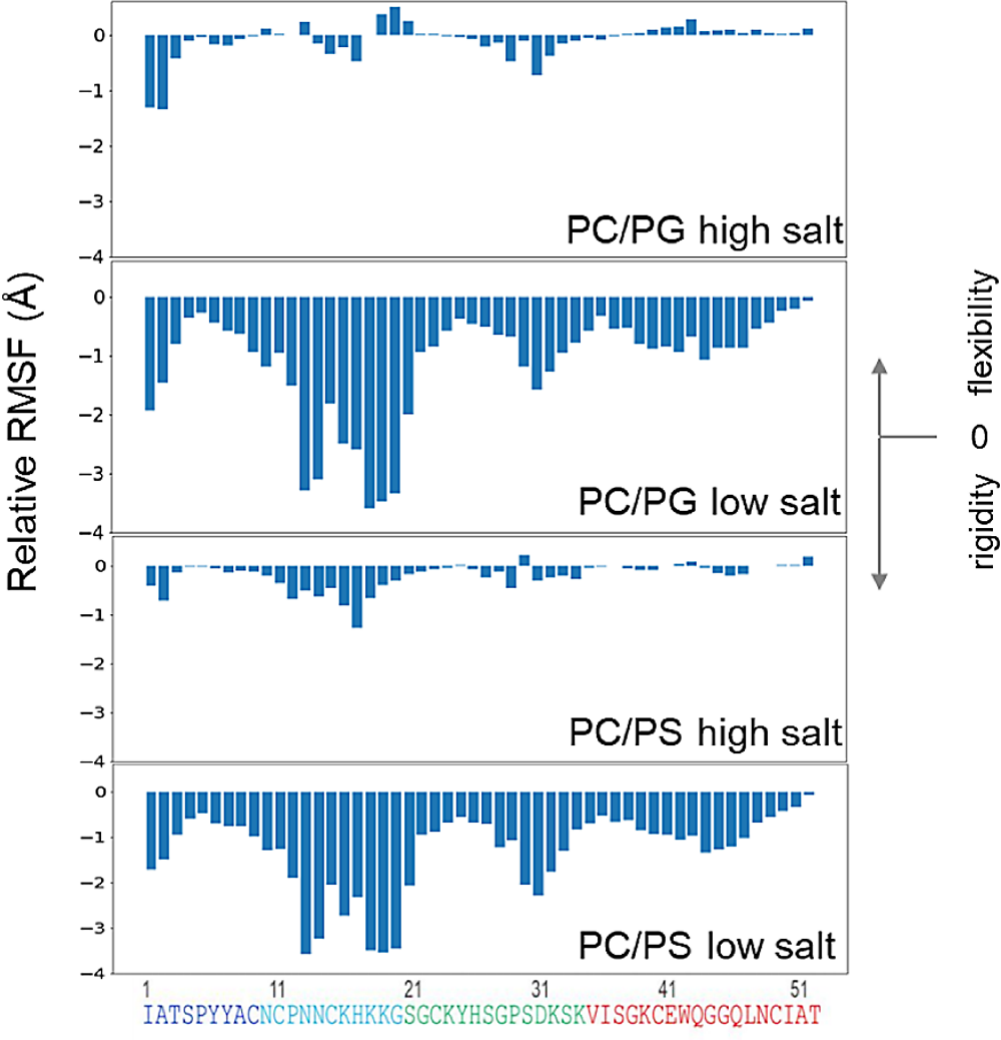
Mapping NFAP2 flexibility upon binding to anionic model membranes,. RMSF changes of NFAP2 upon membrane binding were calculated relative to the fluctuations of the free protein. RMSF values represent the average for the last 500 ns of the simulation in the presence of lipids and for the entire simulation in the absence of lipids. Data shown represent average values of three parallel simulations.

To identify the lipid binding region of NFAP2, we analyzed the per-residue contacts and flexibility for the four anionic membrane systems. To this end, the percentage of lipid contact time was determined in the last 500 ns of the simulations (Figures 5 and S1). The root-mean-square fluctuation (RMSF) values were also calculated in these parts of the trajectories (Figure 6), which report on the flexibility changes upon membrane interaction.

Contact mapping revealed distinct binding preferences (Figures 5 and S1). The flexible region 11–20 involving several cationic lysine residues and the disulfide-bridge segments 11–15, exhibited high contact values in all four systems tested. This general observation suggests that this region could be a key segment in driving the lipid binding of NFAP2. However, in addition to the flexible region, segments 21–26 also made contacts with the membrane in each simulation, with particularly high contact values for Lys24. The N-terminal part, regions 1–8, also contributed significantly to the binding, which was more pronounced with PC/PG compared to PC/PS and at low versus high salt concentrations. Furthermore, contacts with regions 30–39 were also observed in most of the simulations, however, with the highest contact values for the cationic residues, Lys32, and Lys34, again. In two out of the three simulations for the PC/PG low salt system, regions 41–47 also showed contacts, with the highest score for the sole tryptophan, Trp42. The C-terminal residue, Thr52, was also detected in the contact map of all four of these systems, where the low values suggested rather weak contacts with PC lipids. With PC/PG, more contacts are formed with segment 1. PC/PG at low salt concentration showed the less common binding pattern, with extra contacts forming with segment 4 at the expense of contacts with segment 3.

Contact mapping revealed high scores for the cationic lysines making definite contacts with anionic lipid components, with typical contact values of 30–50%. Even higher values were found for most of the lysines to PC, but this does not indicate preferred binding to the neutral PC but likely a result of the fact that PC is 4-fold more abundant in the PC/PG and PC/PS bilayers. Note, however, that the protein showed no affinity to bind to the pure PC as mentioned above. In line with these findings, contact analysis also revealed that PG or PS is typically enriched in the vicinity of the bound protein (Figure S2 and Table S2), highlighting a potential sequestering role for the anionic lipid species driving the interaction. Specifically, while PG or PS constitute only 20% of the lipids in the bilayer, they could be overrepresented by up to ∼50% at the protein binding site, as clearly illustrated by the increased relative contact ratios for these lipids (Table S2).

RMSF analysis results (Figure 6) are consistent with the findings from the contact mapping above. Expectedly, at low salt concentration, we observed an overall increased rigidity for the whole protein in both PC/PG and PC/PS systems compared to its highly flexible nature in water. In particular, flexibility reduced remarkably (by >1 Å) for several protein regions such as for the N-terminal few residues, the segment 11–21 residing within the flexible N-terminal part, and the segment 28–33, the long loop connecting strands β1 and β2. Compared to the very similar patterns observed for PC/PG and PC/PS under low salt conditions, significant differences were found at high salt concentration, where the overall flexibility variations were also much smaller. For PC/PS at low salt concentration, the flexibility/rigidity pattern was comparable to that at high salt concentration, with the most notable loss of flexibility detected for segment 11–21, also identified as the binding region in the contact analysis. By contrast, some residues (13 and 19–21) of the same region showed even enhanced mobility when bound to PC/PG, and the stretch 14–17, including Cys15 followed by two lysines, showed flexibility loss. The reason for the apparent discrepancy could be the difference in the binding mechanism exerted on the two bilayers. While this region is located on the surface of the PC/PS bilayer, forming stable contacts with surrounding lipid head-groups, it might actively move to find a way for inserting into the PC/PG membrane. Furthermore, the highest flexibility loss detected for the N-terminal residues suggests an important role for this protein part under high salt conditions.

For all the simulations above, the starting protein position was a random orientation relative to the membrane, where, in fact, the C-terminal part (segment 4) pointed toward the lipid bilayer. Nevertheless, contact analysis highlighted that the C-terminal segment is rather inactive in binding. Based on this finding, we also analyzed the initial protein–lipid contacts for 100 ns after the first contact was detected. Results indicated that several initial binding patterns could form, irrespective of the lipid system or ionic strength (Figures S5 and S6).

#### 3.2.4 Binding Modes of NFAP2 to Anionic Lipid Bilayers

**Figure 7:**
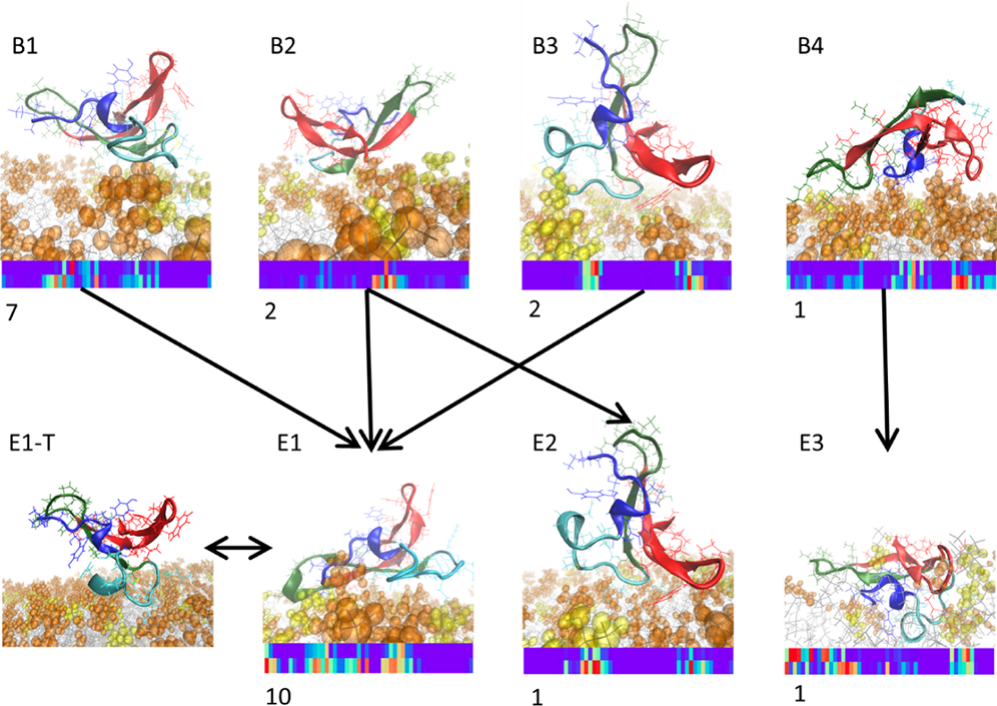
First membrane contacts and final binding modes of NFAP2 to anionic model membranes. Representative snapshots of the various binding modes are displayed, highlighting the orientation of NFAP2 relative to the membrane surface. TOP: Initial binding modes were identified (B1–B4). BOTTOM: End bound states (E1–E3). Note that E1 may flip to a temporary bound state with less direct contact with the membrane (E1-T). Sequential regions participating in binding are displayed under each binding mode similarly to that in Figure 5. For each initial (B) and end (E) bound state, the frequency of occurrence out of the total 12 negative membrane model simulations is displayed. NFAP2 is colored according to its structural segments as introduced in Figure 3. Lipid head-groups of PC and PG or PS are orange and yellow, respectively, and acyl chains are gray. For more details see Section 3.2.4 and Figure S4.

Combining simulation results from the contact mapping and flexibility analysis, we could identify a few favorable membrane binding routes and modes for NFAP2 (Figure 7). It is apparent that binding modes could be shared among the various lipid systems, while alternate binding modes could also form under the same conditions.

We identified four distinct starting orientations for how NFAP2 initiates binding with bilayers. These were categorized for each of the 12 negative membrane runs based on the first 100 ns contact maps after the first contact was detected (Figure S6). The most frequently seen initial binding mode (B1) is similar to the most commonly seen adsorbed end state (E1), although additional key contact regions occurred for the latter (Figure 7). This final binding pattern involves contacts with regions 1–3, 11–26, and 30–39, comprising the N-terminal few residues, the flexible segment 2 with the C11–C15 bridge, strand β_1_, parts of loop L_1–2_, and strand β_2_, respectively. E1 mode was observed for 10 out the 12 individual simulations, which thus occurred for all four different simulation setups. Note that slight differences could be observed between these 10 final binding modes, for instance the N-terminal (region 1–3) can be very inactive sometimes, as we observed for some simulations under PC/PS high salt conditions. In this binding mode, segment 2 is either bound or partially inserted into the membrane, typically between the lipid head-groups. Interestingly, despite E1 being the most observed end state, it could switch temporarily, for short periods of 10–15 ns, to E1-T, where only segment 2 makes contacts with the bilayer, highlighting its previously observed main role in the binding process (Figure 7).^8^

In addition to B1, three additional distinct initial binding patterns could be observed, B2–B4, occurring a total of 2, 2, and 1 times, respectively. B2, involved activity from segment 3, segment 4, and a little from segment 2. This binding mode could either transition into the most commonly seen end state E1 or to E2, where NFAP2 made contacts with the bilayer only with segments 2 and 4, while segments 1 and 4 stayed far above the membrane. For E2, the stabilizing effect comes from segment 4, particularly from Trp42. After the initial binding B3, simulations transitioned to E1, where again Trp42 played an anchoring effect when making the first membrane contact. Considering the relevance of Trp42, a further analysis addressing its relevant positioning was performed by calculating its solvent accessible surface area (RSA). Accordingly, RSA values indicated partially buried Trp side chains both in the presence and absence of lipids (Figure S3 ∼60–75%). This suggests that the Trp is located rather on the membrane surface rather than being inserted deep into the lipid bilayer. Indeed, Trp was mostly observed as surrounded by lipid head-groups in the simulations. B4 and the accompanied end state E3 are both unique. For B4, the end part of segment 4 contributed to most of the initial binding. As for E3, the whole N-terminal part, residues 1–19, lies on the membrane surface; however, with no constraining lipid contacts for segment 2, which enables its active movement toward an inserted state, a crucial step in pore-forming activity observed on *Candida* cell.^7,8^ We note here the stable contacts of segment 1 with PG head-groups, anchoring this flexible part in a rather extended conformation to the bilayer. Furthermore, regions 42–47 from segment 4 are actively making contacts (Figure 7). Although E3 occurs only once out of the 12 simulations, it is the only bound mode which has a deeper insertion into the membrane, thus it could have major relevance when, i.e., oligomerization processes may occur leading to membrane openings as seen earlier.^8^

### 3.3 Experimental Results Support Lipid Binding Preference of NFAP2

To validate computational results, the membrane binding of NFAP2 was also probed experimentally by applying model vesicles with the same compositions as used in the MD simulations (PC, PC/PG, and PC/PS). To test the potential effect of ionic strength, binding assays were carried out in standard PBS (10 mM phosphate with added ∼150 mM NaCl), representing high salt conditions, and low PBS where PBS was diluted with water (typically 20×), representing low salt conditions. The impact of the liposomes on the NFAP2 structure and assembly, as well as the effect of NFAP2 on the integrity of the model vesicles, were addressed.

#### 3.3.1 NFAP2 Triggers the Aggregation of Anionic Liposomes under Low Ionic Strength Conditions

**Figure 8:**
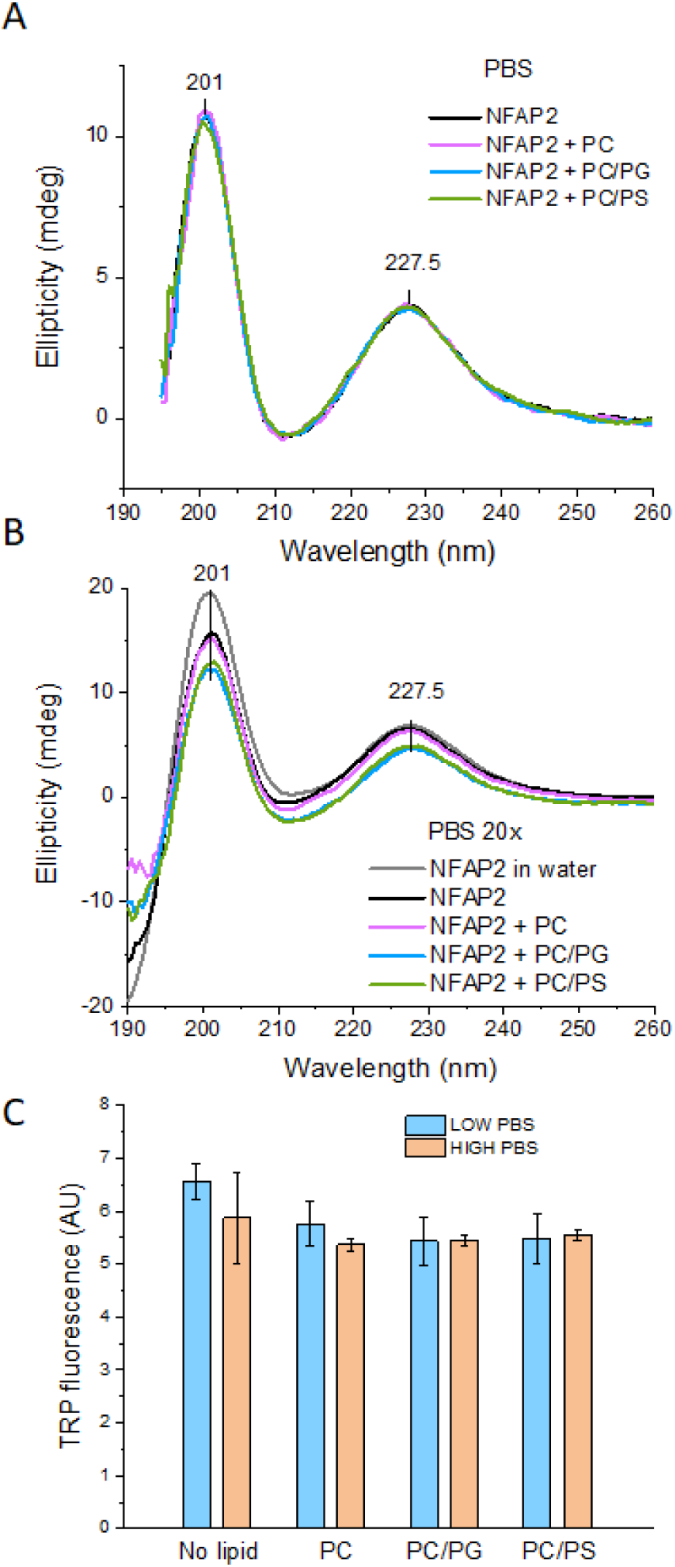
Effect of model vesicles on the solution structure of NFAP2. A,B) Far-UV CD spectra of NFAP2 (25 μM) in the presence and absence of liposomes (635 μM) in PBS, low PBS (20×), and water. Significant spectral variations were observed only with the anionic liposomes in low PBS. C) Intrinsic peptide fluorescence of NFAP2 (5 μM) in the presence and absence of liposomes (125 μM) in low PBS (100×) and PBS.

Membrane binding was assessed first via spectroscopic methods that monitored the protein structure. The solution structure of NFAP2 (Figure 3) revealed that the protein contains a C-terminal folded part constituted of three antiparallel β-strands, whereas the N-terminal half is flexible/disordered. The 3D structure is stabilized by three intramolecular disulfide bonds. Accordingly, the far-UV CD spectra of NFAP2 in aqueous solutions displayed an intense maximum at ∼201 nm assigned to the β-fold of the protein, and a less intense maximum at 227.5 nm, which was attributed to the disulfide bonds.^39^ Consistent with the NMR structure, our CD analysis yielded ∼50% antiparallel β-sheet content, ∼10% turn, and ∼40% disordered fraction as calculated from the spectra collected under high and low salt conditions (Figure 8A,B and Table S3).

At higher ionic strength, the addition of liposomes induced no spectral changes (Figure 8A). In contrast, subtle but characteristic CD pattern variations were observed at low ionic strength selectively with the negatively charged vesicles (Figure 8B). These changes did not affect the position of the two main peaks, but the amplitude of both slightly reduced, as observed by a down-shift of the spectrum by ∼1–3 mdeg between 200 and 240 nm. This spectral variation cannot be attributed to marked conformational changes but rather to light scattering effects due to the aggregation of the vesicles, which was indeed clearly visible upon mixing the components. The protein-assisted vesicle aggregation was confirmed by DLS, detecting micrometer-sized particles with PC/PG and PC/PS. This contrasts with the diameter of individual liposomes of 100 nm, which was measured for NFAP2 with PC (Figure S7 and Table S4).

It should be noted that the intensity of the main peak at 202 nm, unlike that of the peak at 227.5 nm, was found to respond sensitively to ionic strength changes (compare spectra in water vs PBS 20× or PBS, Figure 8A,B). This can suggest either subtle rearrangements within the β-sheet motif or likely oligomerization of the protein under higher salt conditions. This feature is of functional relevance as oligomer formation might affect membrane activity via facilitating protein assembly needed for pore formation. Note that this aspect requires thorough additional investigation with committed MD simulations specifically set up to address this question, a direction that is beyond the current focus of this study.

#### 3.3.2 The Membrane Environment Induces Subtle Variations in Intrinsic Protein Fluorescence

Seeking NFAP2 segments interacting with the lipid membrane, MD simulations, particularly with PC/PG at low salt concentration, highlighted that the sole tryptophan, Trp42, located in the β-core of the protein, could also contribute to lipid binding. This possibility was tested experimentally by monitoring intrinsic Trp fluorescence, which is sensitive to the polarity of the Trp microenvironment.^40^ It is known that a fully water-exposed Trp in monomeric, disordered peptides, and in unfolded proteins show maxima up to 355 nm, while Trp residues within the hydrophobic interior of folded globular proteins or membrane environment could exhibit significantly blue-shifted maxima, even down to ∼330 nm.^41,42^ Accordingly, the emission maximum at 341 nm found for NFAP2 in aqueous solutions is indicative of partially solvent-exposed Trp. This agrees well with the calculated relative RSA values above. In the presence of model vesicles, no shift of the maximum was observed, suggesting no significant changes in Trp environment polarity, presumably due to the lack of deep insertion of the Trp side-chain into the lipid bilayer. Indeed, in the simulations, the indole ring was observed to lie on the membrane surface contacting both PC and PG head-groups (Figure 5). However, quenching of the intensity in the membrane environment was found at both low and high ionic strength (Figure 8C). In high PBS, the addition of liposomes decreased the fluorescence signal with all three liposome compositions applied to the same extent by ∼5–7%, while in low PBS, the somewhat higher quenching effect with PC/PG and PC/PS vs PC (16–17% vs 12%, respectively) could be indicative of more intense interaction of NFAP2 with the anionic lipids.

#### 3.3.3 Selective Variations of the NFAP2–Lipid Interaction Under Crowding Conditions

Binding selectivity was further investigated using the ATR FT-IR technique on dry film samples, analyzing both protein and lipid bands. It should be noted that due to this setup, spectral changes could report on how the interactions formed in the diluted solution can change in the crowded milieu of the dry film, both of which can represent physiologically relevant conditions.

**Figure 9:**
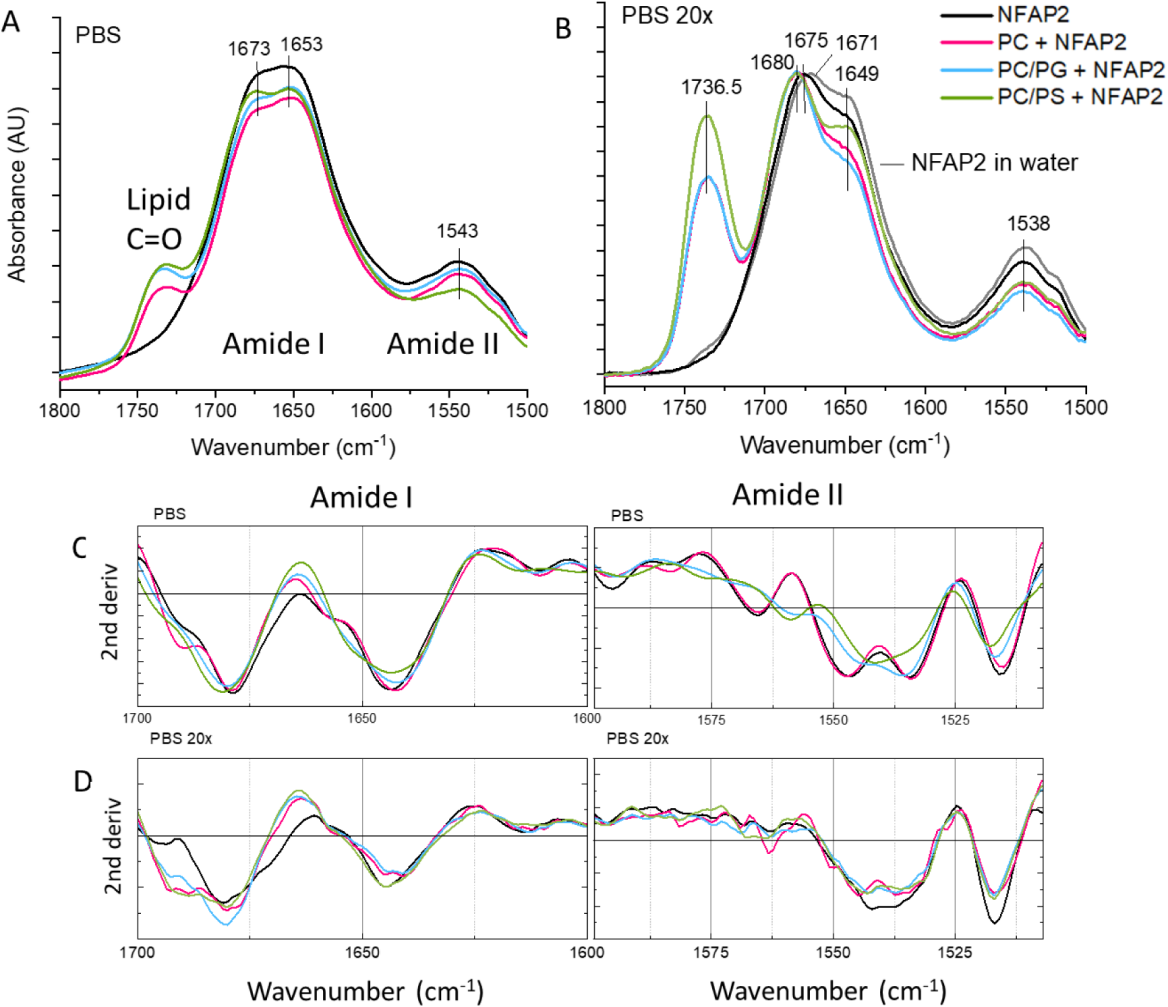
Effect of model vesicles on the NFAP2 structure in dry film. (A,B) Protein amide I and amide II bands (along with lipid C=O band) of IR spectra recorded for dry film samples prepared from solutions containing NFAP2 (25 μM) in the presence and absence of liposomes (635 μM) in PBS and low PBS (20×), or water. (C,D) Second derivative spectra of bands amide I and amide II in PBS and low PBS. The legend given in (B) applies to panels A–D.

In the presence of model vesicles, selective variations were found for the protein bands under both high and low salt conditions (Figure 9). In PBS, the addition of the anionic liposomes resulted in an overall shift to higher wavenumbers observed for all amide I and amide II band components (Figure 9A,C). These spectral variations could be attributed rather to the more oriented positioning of the protein to oriented, polarized lipid bilayers forming on the ATR surface. In contrast, only subtle changes limited to the flexible protein part could be detected with neutral PC vesicles, which agrees with the selectivity to anionic membranes observed in simulations. In low PBS, variations are more distinct (Figure 9B,D). The most remarkable change is the enhanced intensity of the band component at ∼1685 cm^–1^ observed with all lipid vesicles applied, which suggests that the flexible half of NFAP2 might be perturbed upon lipid binding. Interestingly, such an extra shoulder emerging at higher wavenumbers next to the main component has been reported for several cationic AMPs, which could be assigned as a marker for the membrane-bound state.^27,43,44^ Nevertheless, according to the subtle changes of the band at 1645 cm^–1^, the H-bonding network within the β-fold was only slightly affected, which is in line with nonsignificant changes of the protein fold and also agrees with the CD and the MD results above.^45^

Protein–lipid contacts were further assessed by analyzing lipid vibrational bands corresponding to headgroup phosphates, carbonyl neck, and acyl chain regions.

**Figure 10:**
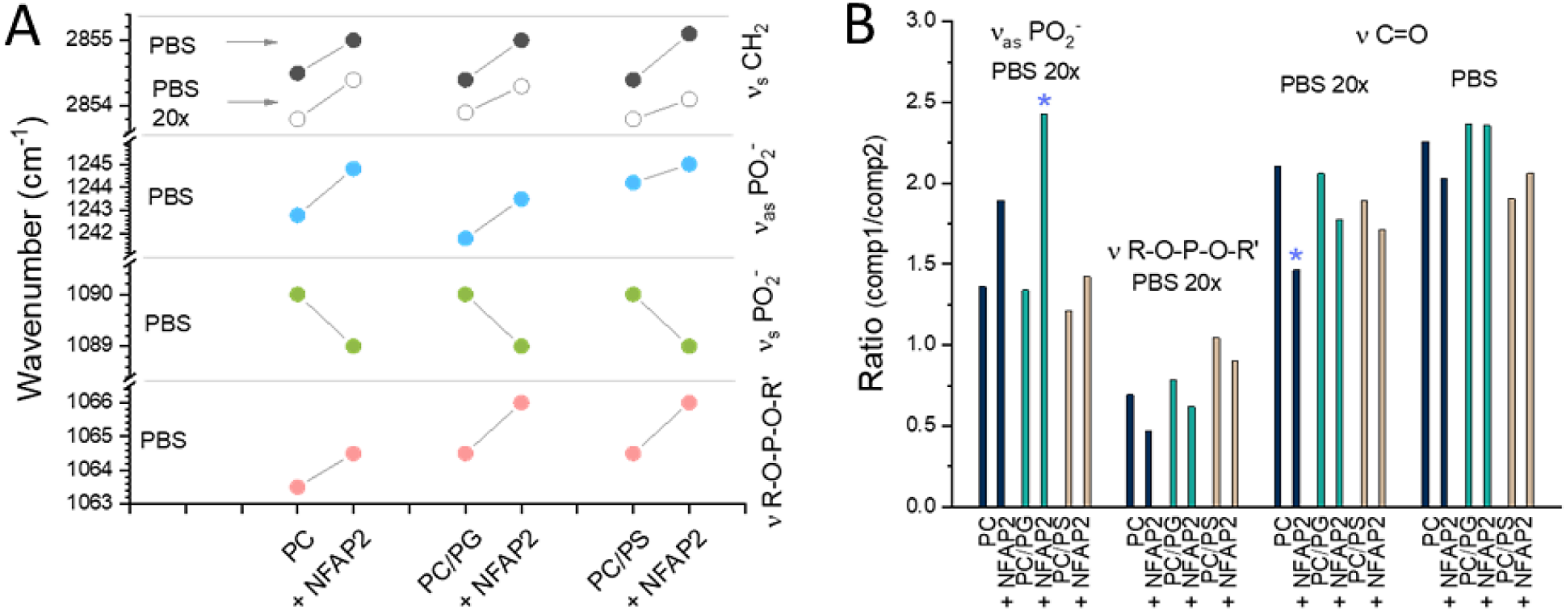
Impact of NFAP2 on model vesicle integrity. Vibrational bands of the three main lipid parts corresponding to the acyl chain, ester neck, and headgroup phosphate regions were analyzed. IR spectra were recorded for dry film samples prepared from solutions containing NFAP2 (25 μM) in the presence and absence of liposomes (635 μM) in PBS, low PBS (20×), or water. NFAP2 induced (A) shifts of individual band components or (B) variations in the relative intensity ratio of band components (comp1 and comp2, for more details see Figures S8–S10).

With all three liposome compositions tested, notable shifts to higher wavenumbers were detected for the methylene vibrational bands at both high and low concentrations of PBS (Figures 10A and S8), which indicates perturbation of lipid order, likely toward looser packing upon contact with NFAP2. It should be noted, however, that perturbations of this region are not necessarily related to the insertion of the protein deep into the hydrophobic interior but could be mediated by binding events in outer membrane regions.

Variations were also observed for the phosphate bands (Figures S9 and 10A). In high-phosphate PBS, delicate shifts (Figure 10A) indicated perturbation of the lipid headgroup region in the presence of NFAP2. By contrast, in low PBS, rather uniform changes were found for the νR–O–P–O–R’ vibration with all three model membranes, where the ν_as_PO_2_^–^ band was affected more selectively, indicating the highest and lowest perturbation for PC/PG and PC/PS, respectively.

Interestingly, for the lipid C=O band representing the lipid neck region, the most remarkable change was found with PC at low ionic strength, while the anionic lipid bilayers were less significantly affected upon the addition of the protein. In contrast, the perturbation in PBS followed a different trend, with small but opposite effects for PC and PC/PS, respectively, and no change for PC/PG (Figure 10B).

Summarized, lipid vibration data suggested various binding modes for NFAP2 under different membrane conditions, leading to the perturbation of various levels and layers of the lipid bilayer. This finding, in general, agrees well with the nonuniform binding behavior observed in MD simulations. The highest effects point to the binding of NFAP2 to headgroup phosphates on PC/PG vesicles, while to the lipid neck region on PC vesicles (Figure 10B, marked with stars). In comparison to headgroup contact analysis from simulations (Figure 5), NFAP2 interaction with lipid phosphates could also be detected experimentally. In contrast, less pronounced spectral effects could be detected for these lipid regions on the PC/PS membrane, likely due to the complicated charge pattern of PS (Figure 2), which could allow for the distribution of various NFAP2 contacts along its spacious headgroup. In this case, as IR spectra seem to be less useful in detecting binding to the PC/PS surface, one could primarily rely on simulation results.

## 4 Summary and Outlook

The all-atom simulations demonstrated a selectivity of NFAP2 to lipid bilayers mimicking negatively charged fungal/bacterial cell membranes. This selectivity could be mediated primarily by anionic lipid components, even if nearby PC species in two-component PC/PG or PC/PS bilayers could actively contribute to binding. Moreover, the lack of strong binding to neutral lipids clearly demonstrated in simulations, is in line with the qualitative observations of experimental lipid strip assays, and might partially explain the low toxicity of NFAP2 observed in human cells.^7^ The obtained MD trajectories also highlighted the rather promiscuous binding nature of NFAP2, which showed that the membrane-bound state could be reached via various binding routes (Figure 7). Nevertheless, in the commonly observed binding mode(s), NFAP2 resides on the membrane surface anchored by contacts via its flexible N-terminal part. Particularly, the region 10–20 shows a potency to insert into the lipid bilayer, whereas segments of the β-fold can also contribute to stabilizing the bound state. This has a direct connection to antifungal activity of NFAP2, as the sequence region 13–23 was shown to exhibit antifungal activity alone as well.^8^ Note, one unique contribution of several MD simulations to atomic level insight is that these also revealed key roles for other residues, including Lys24, Lys32, Lys34, and Trp 42. Furthermore, the transient process of initial lipid binding could also be captured by MD runs, based on which the importance of several regions could be deciphered, which is highly relevant for further NFAP2-based developments.

The impact of the actual ionic strength on the binding affinity was also demonstrated in the simulations, as a stronger membrane interaction, i.e., more stable contacts, was found in general under low salt conditions.^46^ The latter finding implies that electrostatic attraction plays important roles in the interaction of NFAP2 with anionic membranes, which is further supported by the cationic nature of the main binder segment 10–20. Likewise, such a salt dependence has been observed for cationic AMPs as well, pointing to key electrostatic interactions upon membrane binding.^47−50^ Note, however, that for PC bilayers, no binding was found irrespective of the salt concentration, and for the anionic systems, the simulations resulted in similar binding modes but with different salt conditions. These results overall suggest that the relative importance of the salt conditions is also dependent on the particular lipid composition.

Experimental findings closely supported most of the *in silico* observations, despite the fact that experimental and computational setups differ in several key aspects, from the intrinsic vesicle curvature through liposome aggregations to the potential AFP oligomerizations in experiments. Compatible with MD results, experimental data also suggested that the lipid binding of NFAP2 is sensitively affected by environmental factors like ionic strength. Selective effects exerted on or induced by anionic membranes were detected under both high and low salt conditions; however, these manifested in different ways compared to the simulations. Notably, contacts between NFAP2 and PC head-groups were detected to form in simulations, which is reasonable due to the anionic phosphate group residing in the zwitterionic PC, too. In accordance, here IR analysis also pointed to a different binding mode of NFAP2 on PC in comparison to anionic membranes, affecting the lipid carbonyl region of PC.

In experimental conditions, both protein halves showed the ability to interact with the membrane, while the overall protein conformation was not perturbed markedly. This agrees with the computational results and could readily be linked to the stabilizing effect of the disulfide bridges.

Structural data obtained here are also consistent with previous CD measurements, showing no significant alteration of the protein structure upon treatment of fungal cells.^7,39^ This feature is in contrast with some β-defensins with a similar β-fold, where the flexible C-terminal part folds into an amphipathic helix upon interaction with anionic lipid bilayers.^51^ For NFAP2, some ability to fold into a helix was indicated, as segment 5–9 was assigned as a short helical motif in the solution NMR structure (Figure 3). By contrast, the helical wheel representation of the N-terminal half (Figure S11) suggests that folding to an amphipathic helix is not favored. In this conformation, the three lysines and the hydrophobic residues of this region would point to rather random directions around the helix axis (Figure S11). Moreover, the disulfide pattern could also interfere with the formation of a regular helix. Instead, the C11–C15 disulfide bridge defines a “fingertip”, which can act as an insertion motif. In this mechanism, the three lysines next to this fingertip, Lys16, Lys18, and Lys19, can play important roles, acting as an electrostatic Velcro, in line with our MD simulations. This might be a crucial step in the pore-forming activity observed experimentally on *Candida* cells.^7^ Interestingly, the active fragment (corresponding to res 13–23 but missing the disulfide bond between C11 and C15) is exclusively composed of hydrophilic residues except for the two cysteines (Figure S11). Likewise, segment 2 in this study, sequentially close to the active fragment, also lacks hydrophobic side chains other than cysteines (Figure 1). Such a contribution of cysteines to the hydrophobic moment of an AMP sequence has already been suggested for cysteine-enriched AMP families.^52^ These data also point to a membrane disruption mechanism in which the formation of a pore spanning the hydrophobic interior of a lipid bilayer might involve the C11–C15 disulfide bridge.

It was also revealed here for NFAP2 that the N-terminal part could be key to anchoring the protein. The importance of this is in line with the fact that most of the natural AMPs have a free N-terminal amine, contributing to their cationic nature. Elimination of the charge at the N-terminus could result in reduced antimicrobial activity.^53^ Moreover, in some simulations Trp42 was also indicated to have an anchoring-like function, a role well-known for tryptophan residues in common AMPs,^54^ which could stabilize a membrane-bound state even if not inserted into that, as observed for many membrane proteins.

Our findings demonstrated that NFAP2 could effectively bind to lipid bilayers incorporating simple anionic glycerophospholipids. However, under experimental conditions, oligomerization could be part of the molecular toxic mechanism, and it also has to be considered that natural cell membranes contain various sphingolipids and a remarkable sterol content as well. Consequently, further molecular insight with simulations taking into account these aspects is needed to clarify the membrane-disrupting activity of NFAP2, a study currently in progress in our laboratory.

## Data Availability

The refined NMR structure of NFAP2 was deposited to the Protein Data Bank (PDB ID 8RP9) and the Biological Magnetic Resonance Data Bank (BMRB ID 34 891). Membrane models were generated using the CHARMM-GUI Web site.^18^ Molecular dynamics simulations were performed with version 2019.4 of GROMACS.^16^ The CHARMM36m^17^ force field was applied for the simulations. Trajectories were evaluated using the MDAnalysis Python package.^24,25^ Images illustrating results were produced using Visual Molecular Dynamics (VMD).^26^ The input files and coordinate files for the simulations are available at 10.5281/zenodo.10653764.

## Supporting Information Available

Molecular Dynamics parameters (Table S1); analysis of the number of NFAP2–lipid contacts (Table S2); conformational analysis of NFAP2 (Table S3); effect of NFAP2 (Table S4); contact analysis of NFAP2 (Figure S1); analysis of the number of NFAP2–lipid contacts (Figure S2); relative solvent accessible surface area of TRP residues (Figure S3); adsorbed end state of NFAP2 (Figure S4); initial lipid contacts anchoring NFAP2 (Figure S5); adsorbed end state of NFAP2 (Figure S6); effect of NFAP2 on model vesicles (Figures S7–S10); helical wheel representation of NFAP2 segments (Figure S11) (PDF)
