# Supplementary material for: Mapping of the Lipid-Binding Regions of the Antifungal Protein NFAP2 by Exploiting Model Membranes
Source: J Chem Inf Model. 2024 Aug 16;64(16):6557–69. doi: 10.1021/acs.jcim.4c00229 (PMC11351017; doi:10.1021/acs.jcim.4c00229)
Supplement: Supplementary file 1 — ci4c00229_si_001.pdf [file ci4c00229_si_001.pdf]

Supporting Information

Mapping the lipid binding regions of the antifungal protein NFAP2 exploiting model membranes

Olivér Pavela[1,2], Tünde Juhász[1*], Liliána Tóth[3], András Czajlik[4,5], Gyula Batta[4], László Galgóczy[3,6*], Tamás Beke-Somfai[1*]

[1] Institute of Materials and Environmental Chemistry, HUN-REN Research Centre for Natural Sciences, Magyar tudósok körútja 2, H-1117 Budapest, Hungary
[2] Hevesy György PhD School of Chemistry, Eötvös Loránd University, Budapest, Pázmány Péter sétány 1/A, H-1117, Budapest, Hungary
[3] Department of Biotechnology, Faculty of Science and Informatics, University of Szeged, Közép fasor 52, H-6726 Szeged, Hungary
[4] Department of Organic Chemistry, Faculty of Science and Technology, University of Debrecen, Egyetem tér 1, H-4032 Debrecen, Hungary
[5]Department of Biochemistry, Institute of Biochemistry and Molecular Biology, Semmelweis University, Tűzoltó u. 37-47, H-1094 Budapest, Hungary
[6] Institute of Biochemistry, HUN-REN Biological Research Centre, Temesvári krt. 62, H-6726 Szeged, Hungary

Table S1. Molecular Dynamics parameters for production runs

integrator	md
dt	0.002
Nsteps	500000000
cutoff-scheme	Verlet
nstlist	20
rlist	1.2
vdwtype	Cut-off
vdw-modifier	Force-switch
rvdw_switch	1.0
rvdw	1.2
coulombtype	pme
rcoulomb	1.2
DispCorr	no
tcoupl	Nose-Hoover
pcoupl	Parrinello-Rahman
pcoupltype	semiisotropic
constraints	h-bonds
constraint_algorithm	LINCS

Bilayer PC/PG

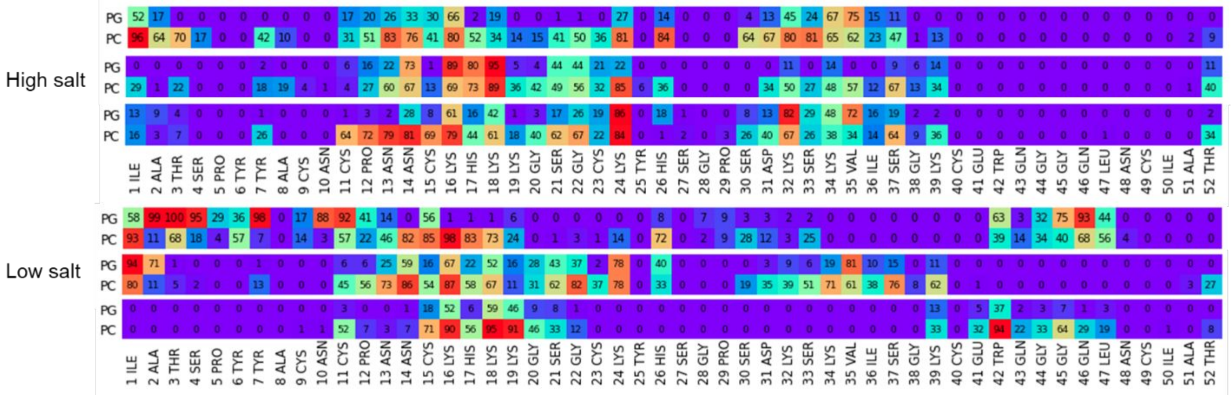

Bilayer PC/PS

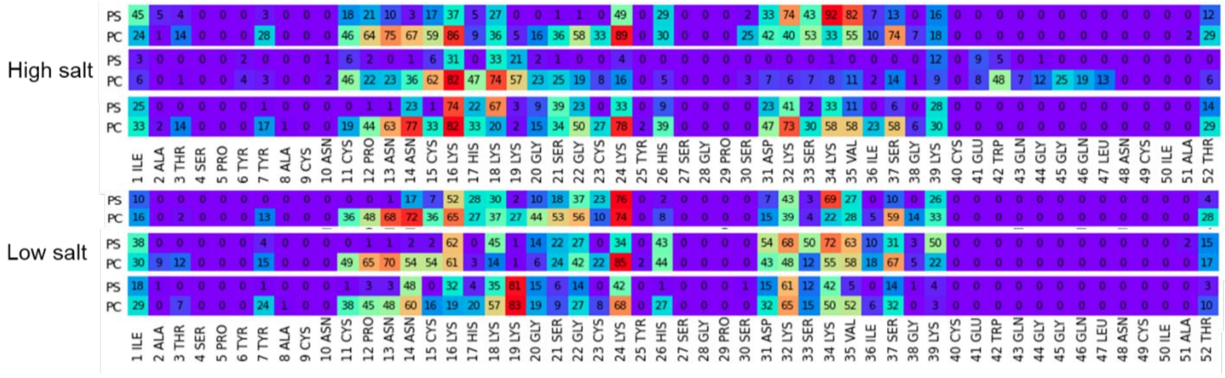

Figure S1. **Contact analysis on NFAP2 when bound to anionic model membranes for the individual simulations.** Values represent the percentage of frames in which contacts formed between any residue of NFAP2 and a particular lipid. Values were calculated for the last 500 frames corresponding to the last 500 ns of the simulations.

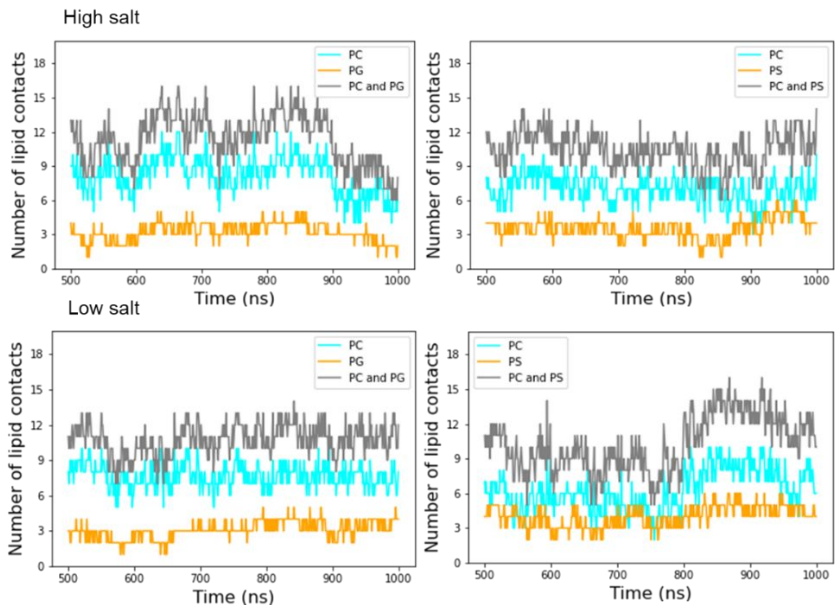

Figure S2. Analysis of the number of NFAP2-lipid contacts in the simulations with anionic bilayers, averaged the 3 parallel simulation runs. The number of NFAP2-lipid contacts are shown for the last or 500 ns. Values refer to the average of three parallel runs.

Analysis showed that

- the total number of lipids contacting a bound NFAP2 shows a variation between 9 and 15,
- for PC/PG, more lipid contacts with less variation are formed at low salt conditions,
- for PC/PG, more contacts form with anionic lipids at low salt than at high salt,
- for PC/PS, more contacts form with anionic lipids at high salt than at low salt,
- for PC/PS at high salt, the number of contacts with anionic lipids is almost equals that with neutral (PC) lipids.

Table S2. Analysis of the number of NFAP2-lipid contacts in the simulations with anionic bilayers. Average PC-to-PS or PC-to-PG ratios of the lipids contacting NFAP2 were calculated for the last 200 ns of the simulations, where NFAP2 is bound to the membrane. The nominal ratio of 4 corresponds to the composition of 80% PC, and 20% PG or PS. Note the significantly reduced ratio for most of the systems indicating that the anionic lipids are overrepresented in the binding site of NFAP2.

Bilayer, condition	Lipid contact ratio			Average
	Set 1	Set 2	Set 3	
Nominal ratio	4.00	4.00	4.00	
PC/PS, high salt	1.39	3.20	1.75	2.11
PC/PS, low salt	2.57	1.16	2.42	2.05
PC/PG, high salt	3.47	2.57	1.88	2.64
PC/PG, low salt	1.60	2.11	4.25	2.65

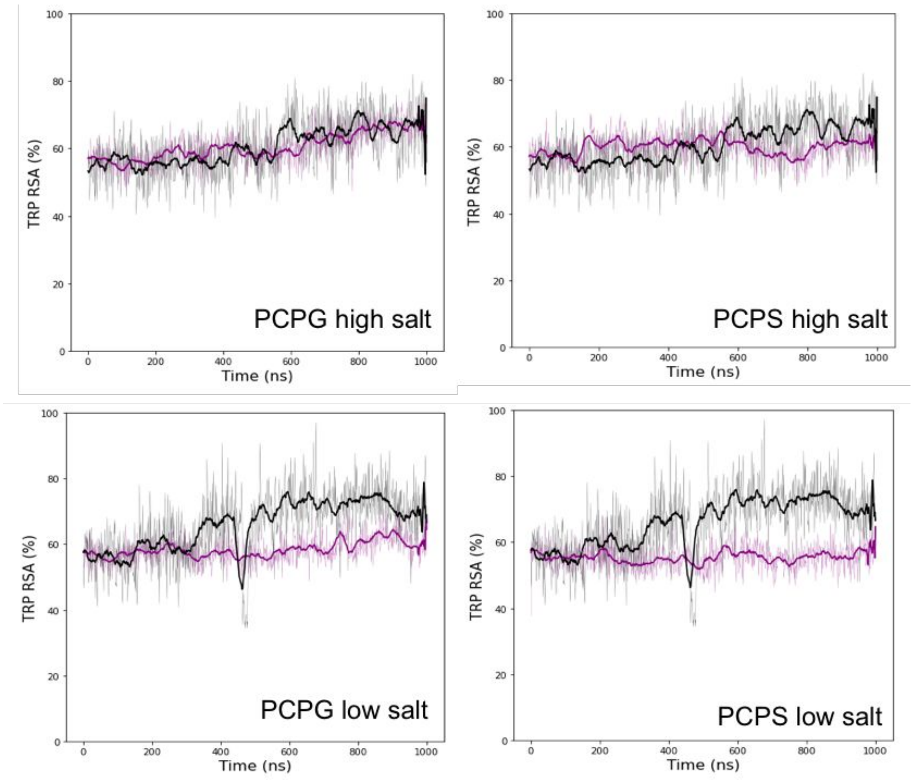

Figure S3. Relative solvent accessible surface area of TRP residue in the presence and absence of anionic bilayers. The maximum solvent accessible surface area (SASA) value was taken as 2.64 nm^2 (Tien. et al 2013), to which the calculated SASA values were normalized yielding the RSA values shown RSA values represent average of 3 parallel runs in membrane simulations (purple), along with reference values for NFAP2 (black) in high salt water for high salt systems, or in low salt water for low salt systems**.**

Note the highly comparable values at about 60% calculated in the presence and absence of lipids at high salt. At low salt after 300 ns, RSA values deviate for the lipid simulations from those for NFAP2 alone, resulting in up to 20% lower values in membrane environment. Interestingly, RSA values remain rather constant at the initial level during the membrane simulation, and the deviation arises from the increasing RSA values observed for the free protein.

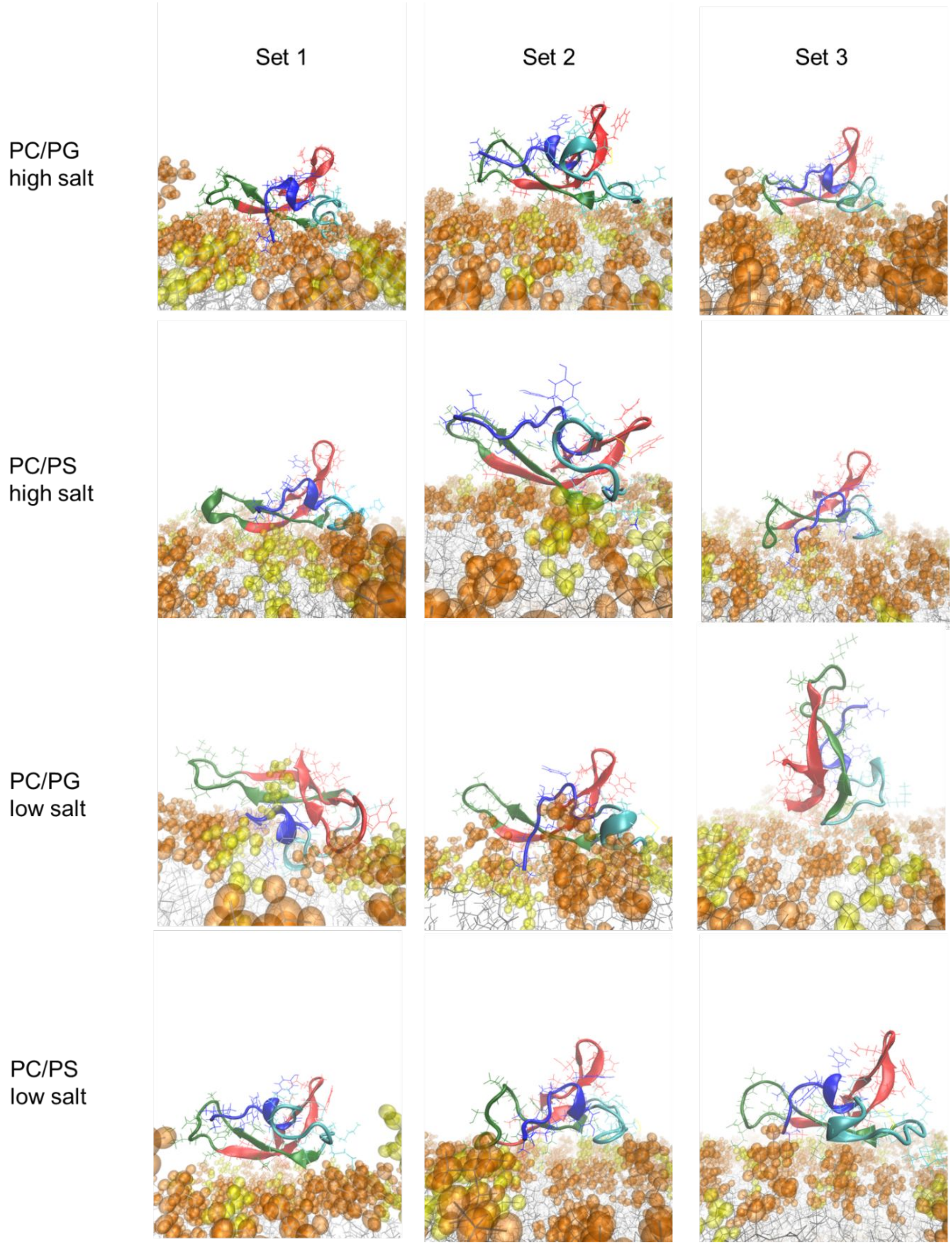

Figure S4. Adsorbed end state of NFAP2 to anionic membranes. Representative snapshots highlighting the relative orientation of the structural regions for NFAP2 were taken at the last frame of the simulations. Simulation set 1on left side, simulation set 2 in the middle, and simulation set 3 on the right side. For PC/PG high salt of set 2, two alternative orientations were found where the relative movements between the two extreme positions are indicated by grey arrows. NFAP2 is colored according to its structural segments as introduced in Figure 3. For the lipids, head groups of PC, and PG or PS are orange and yellow, respectively, acyl chains are grey. Note that segment 4 (red) mostly points away the membrane while segment 2 (cyan) is positioned closest to the lipid bilayer.

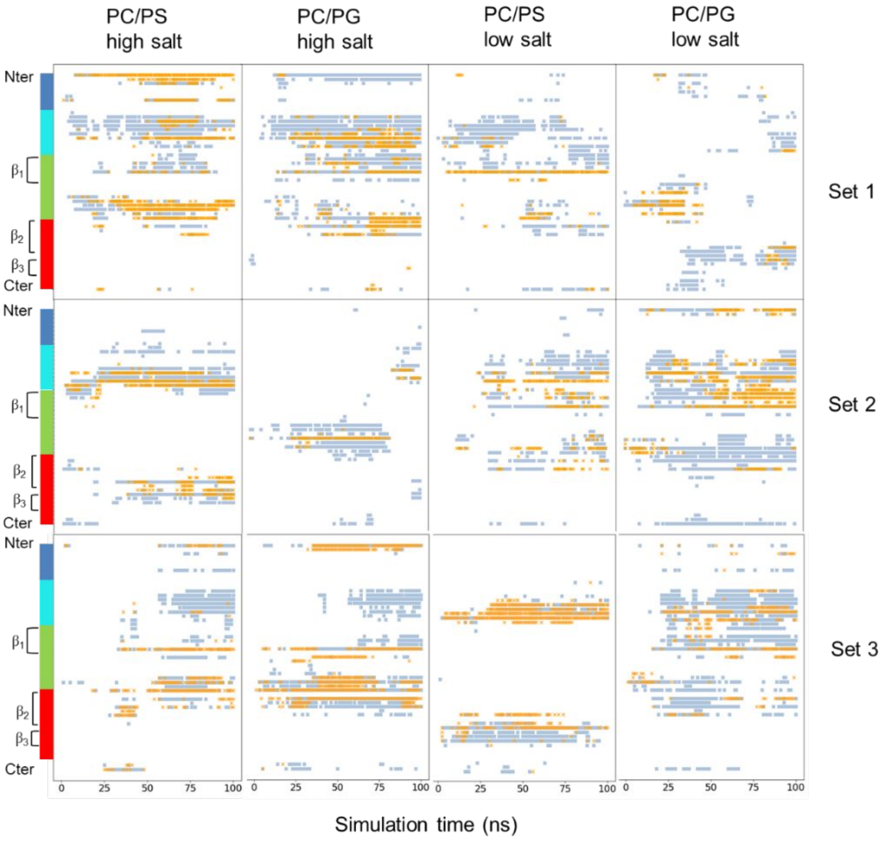

Figure S5. Initial lipid contacts anchoring NFAP2 to anionic lipid bilayers during the individual runs. Lipid contacts were analyzed for 100 ns (100 frames) from the first contact detected for the 3 sets of simulations of the anionic membrane systems. Blue and orange symbols mark contacts with PC and the anionic (PG or PS) moieties, respectively. Four-color stripes indicate the four protein segments from the N-terminus (Nter) to the C-terminus (Cter) as introduced in Figure 3, and the positions of the strands along the sequence are also shown

.

Bilayer PC/PG

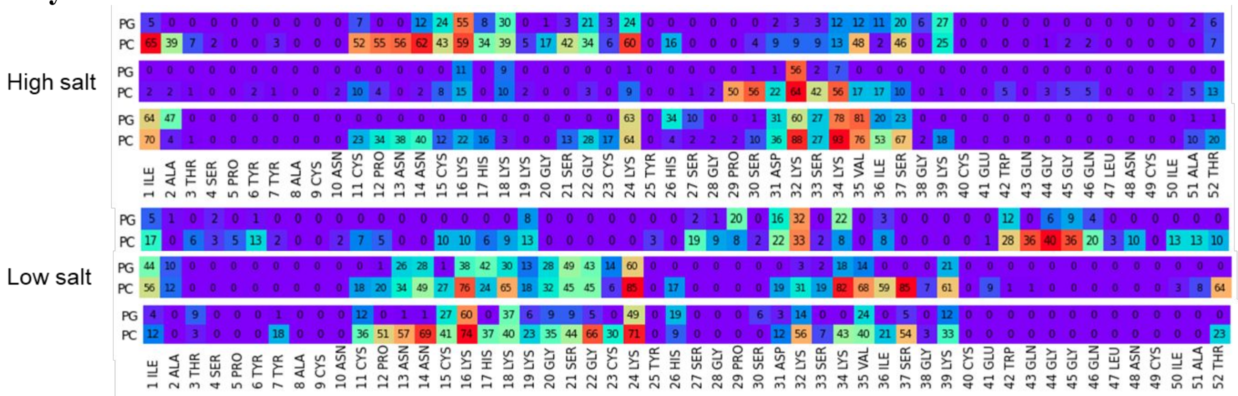

Bilayer PC/PS

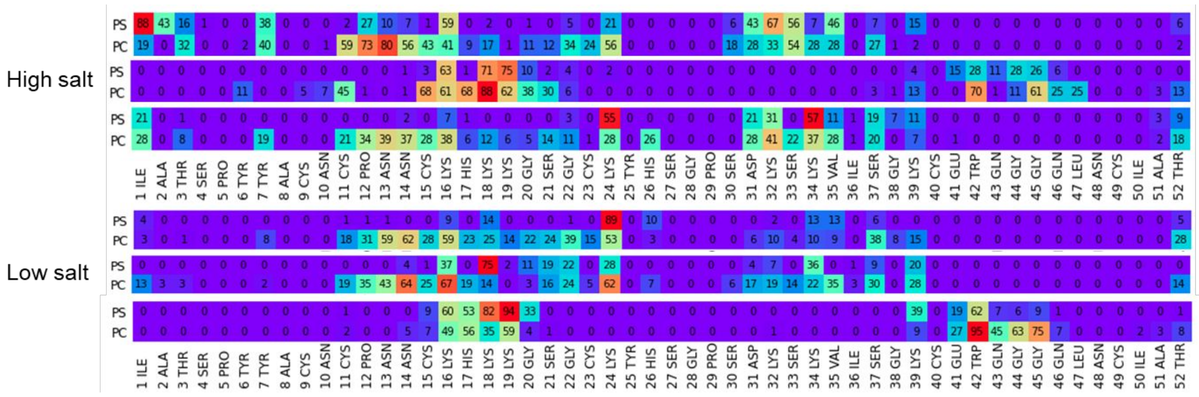

Figure S6. **Contact analysis on NFAP2 upon initial binding to anionic model membranes for the individual simulations.** Values represent the percentage of frames in which contacts formed between any residue of NFAP2 and a particular lipid. Values were calculated for 100 ns (100 frames) from the first contact detected.

Table S3. Conformational analysis of NFAP2 based on CD spectra. The secondary structural element content was calculated using the BeStSel method. As liposome scattering and/or the high chloride ion content results in high absorbance at < 195 nm in PBS, spectra recorded in PBS were analyzed in the 200-250 nm range. Spectra collected in low PBS allowed reliable spectra down to 190 nm, thus were evaluated in the 190-200 nm and the 200-250 nm range. Note the high beta structure content of the protein, preserved upon addition of liposomes.

	Evaluation range	Helix	Antiparallel beta	Parallel beta	Turn	Others
NFAP2 in water	190-250 nm	3	37	0	14	46
NFAP2 in PBS 20x	190-250 nm	1	39	0	14	46
NFAP2 with PC/PG in PBS 20x	190-250 nm	2	42	0	11	45
NFAP2 in water	200-250 nm	8	68	0	10	15
NFAP2 in PBS 20x	200-250 nm	10	64	0	12	15
NFAP2 with PC/PG in PBS 20x	200-250 nm	7	51	0	9	33
NFAP2 in PBS	200-250 nm	4	45	0	12	40

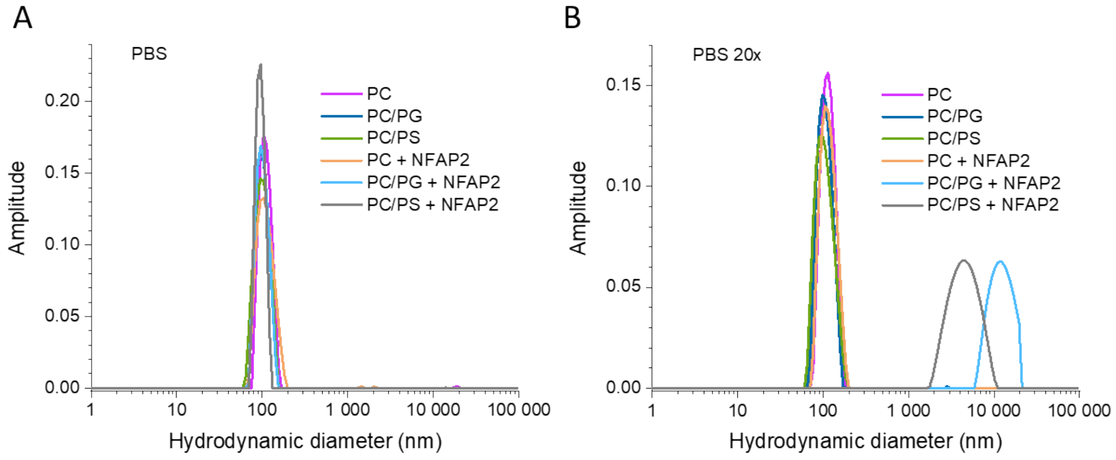

Figure S7. Effect of NFAP2 on the size of model vesicles. Intensity size distribution of the liposomes (635 µM) was determined with DLS upon addition of NFAP2 (25 µM) in PBS (A) and low PBS (B). No significant size variations were observed in PBS whereas NFAP2 induced aggregation of anionic lipid membranes at low ionic strength.

Table S4. Effect of NFAP2 on the size of model vesicles. Diameter and polydispersity (PD) values were obtained from DLS measurements.
NFAP2 was able to induce vesicle aggregation at low ionic strength conditions. The effect was more pronounced at higher NFAP2 and liposome concentrations, leading to the formation of micrometer sized associates. At lower concentrations, the approximately 1.5-fold vesicle sizes suggest bridging of vesicles by NFAP2.

	Diameter (nm)	PD (%)	Diameter (nm)	PD (%)	Diameter (nm)	PD (%)
	PBS		PBS 20x dilution*		PBS 100x dilution**	
PC	108	18	120	18	110	23
PC/PG	100	25	102	25	110	23
PC/PS	100	19	102	24	101	26
PC + NFAP2	108	24	110	21	110 - 119	15 - 29
PC/PG + NFAP2	100	24	>3,000	>60	178	24 - 40
PC/PS + NFAP2	100	18	>1,000	>60	151 - 164	17 - 22

* at 25 µM NFAP2 and 635 µM lipid
** at 5 µM NFAP2 and 125 µM lipid; in the presence of NFAP2 values refer to two sets of measurements

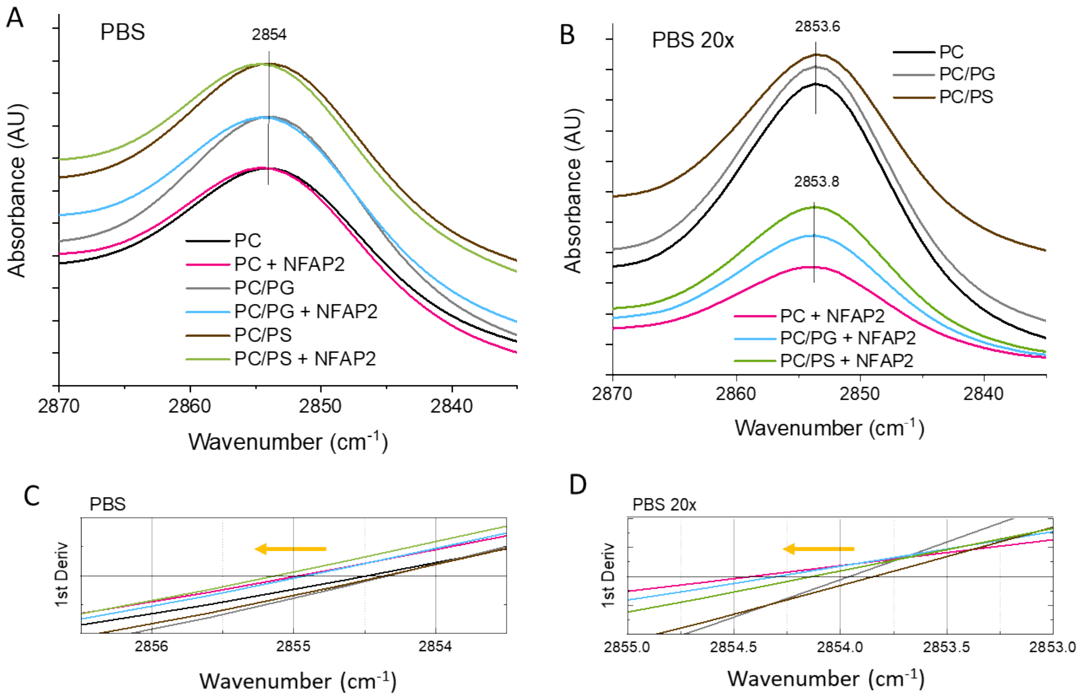

Figure S8. Effect of NFAP2 on the order and packing of model vesicles obtained from ATR IR analysis. Spectra were recorded for dry film samples prepared from solutions containing NFAP2 (25 µM) in the presence and absence of liposomes (635 µM) in PBS (A), or low PBS (20x) (B). Parts of the IR spectra shown correspond to lipid CH_2 vibrational bands (ν_s CH_2). Variations in the CH stretching vibrations reports on the packing and order of the acyl chains. The first derivative spectra (C-D) highlight that the band maxima are shifted to higher wavenumbers upon addition of NFAP2 (marked by yellow arrows). Positions of the maxima are shown in Figure 10A.

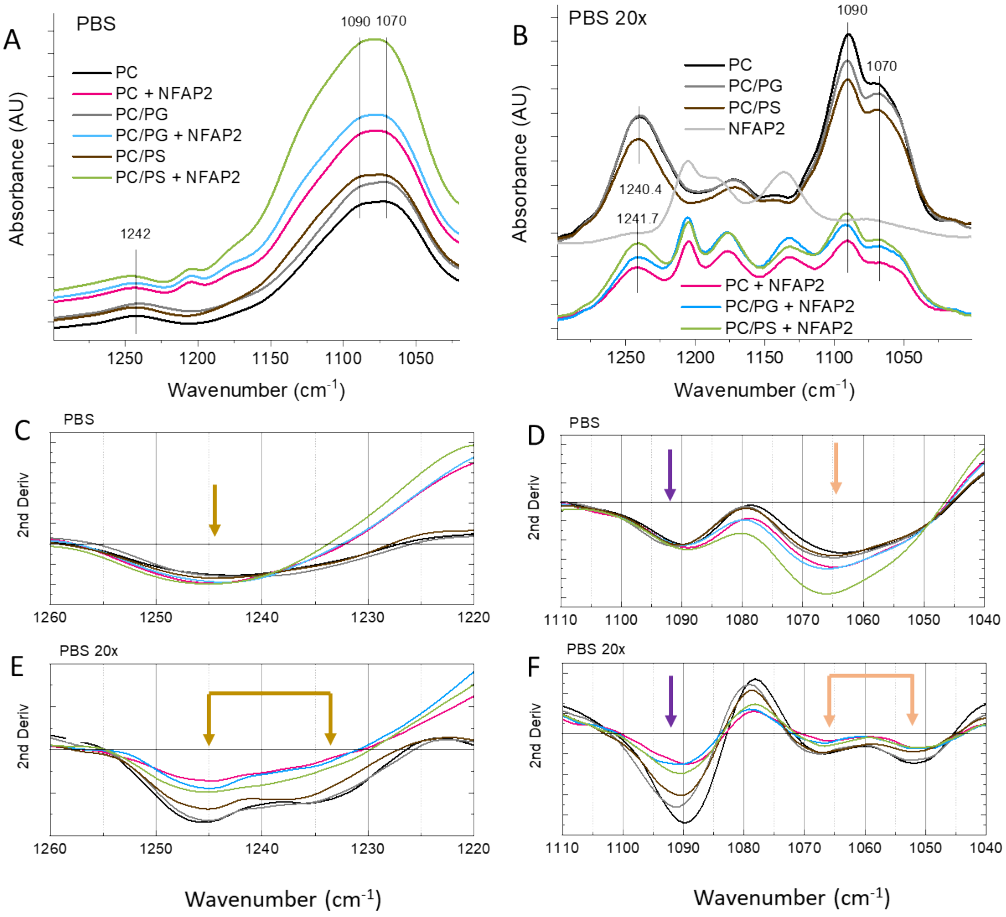

Figure S9. Effect of NFAP2 on the lipid head-groups of model vesicles obtained from ATR IR analysis. Spectra were recorded for dry film samples prepared from solutions containing NFAP2 (25 µM) in the presence and absence of liposomes (635 µM) in PBS (A), or PBS 20x (B). Parts of the IR spectra shown correspond to phosphate vibrational bands, i. e. the symmetric phosphate vibrational bands (ν_s PO_{2-}) at 1230-1250 cm^{-1}, the antisymmetric phosphate vibrational bands (ν_{as} PO_{2-}) at ~1090 cm^{-1} as well as the phosphate ester band (ν_s R-O-P-O-R') at ~1050-1070 cm^{-1}, which are informative of the hydration and conformational state of the lipid head-group phosphate moieties. To highlight spectral variations, the second derivative spectra (C-F) were used for analysis. Note that in high-phosphate PBS, contribution from the inorganic phosphate ions dominates the phosphate vibration region while in low PBS the amount of lipid phosphates and inorganic phosphates is comparable. Due to the latter fact, bands are typically separated to two band components in low PBS, marked by double arrows. Here the two main band components were analyzed for their relative intensity, and ratio values are shown in Figure 10B. In contrast, a single arrow for a band indicates dominance of one main band component that was analyzed for shifts; values derived are shown in Figure 10A.

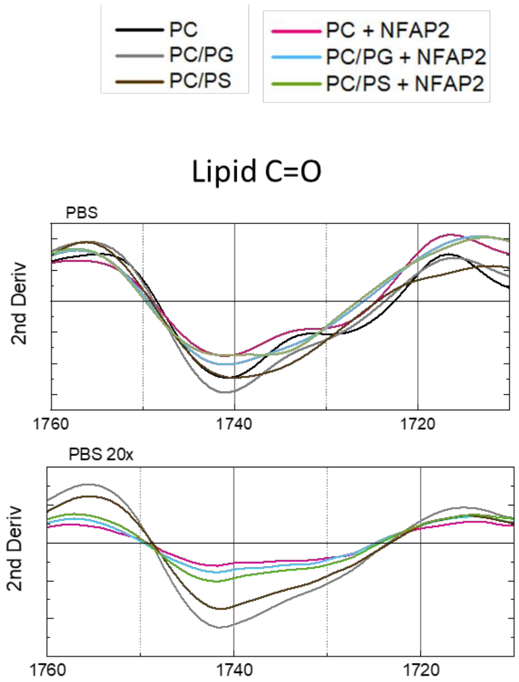

Figure S10. Effect of NFAP2 on the lipid neck part of model vesicles obtained from ATR IR analysis. Spectra were recorded for dry film samples prepared from solutions containing NFAP2 (25 µM) in the presence and absence of liposomes (635 µM) in PBS, or PBS 20x. Parts of the IR spectra shown correspond to lipid ester C=O vibrational bands (ν C=O). Note that these spectral regions can also be seen, along with protein amide bands, in Figure 9. The lipid C=O bands report on the hydration state of the lipid neck region as it is a composite band of the hydrated and non-hydrated lipid ester populations. Accordingly, the relative intensity ratio of the two main components (at 1742 cm^{-1} and 1730 cm^{-1}) in the second derivative spectra were analyzed, and values derived are represented in Figure 10B.

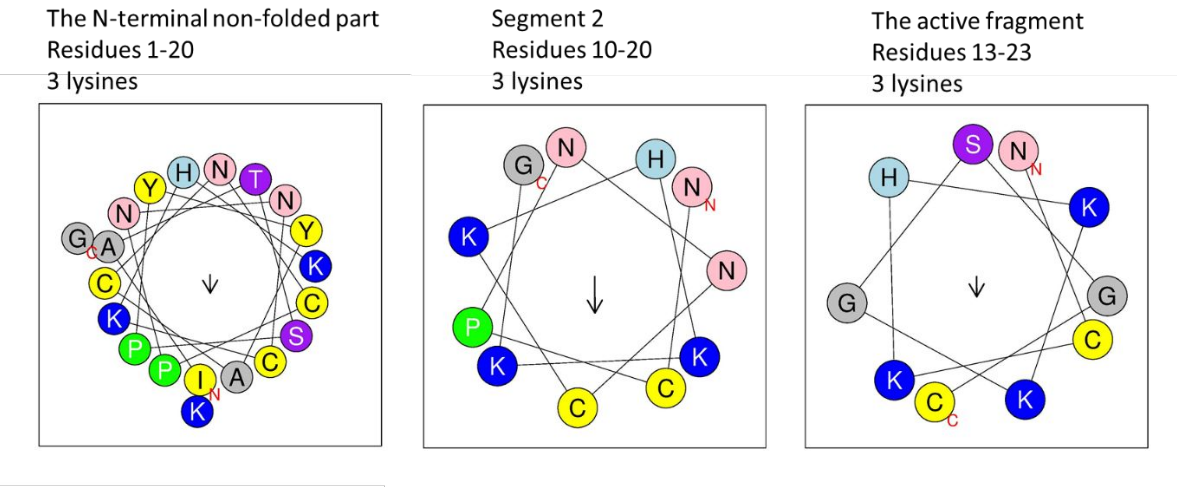

Figure S11. Helical wheel representation of segments of NFAP2. The theoretical helical wheel considering a linear sequence with cysteines not involved in disulfide bonds, was prepared for the N-terminal non-folded part, for segment 2, and the active fragment (for the sequences see also Figure 1). Drawing was done at https://heliquest.ipmc.cnrs.fr/cgi-bin/ComputParams.py.

For the N-terminal non-folded part that the cationic (blue) and hydrophobic (yellow) residues are rather randomly distributed around the helix axis, and the two proline residues are not favored in the helical conformation. Segment 2 and the active fragment are composed of hydrophilic residues, lacking hydrophobic side chains.

Methods

NMR spectroscopy

For NMR experiments ^{15}N- and ^{15}N-/^{13}C-labeled $NFAP_2$ samples were prepared in 20 mM acetate buffer (5% D_2O) at pH 4.5. The protein concentration of latter sample, used mainly for the structure calculation, was 375 μM. The NMR spectra were recorded on Bruker Avance NEO (700 MHz) spectrometer (Billerica, MA, USA) at 298 K temperature. The NMR signal assignment and structure determination was carried out the same way as described for PAFC, our previous antifungal disulfide protein [1]. Briefly, the standard three-dimensional triple-resonance experiments [HNCO, HN(CA)CO, HNCA, HN(CO)CA, HNCACB and HN(CO)CACB] were used for the identification of the protein backbone atoms and sequential resonance assignments. For side-chain resonance assignments the HC(C)H COSY, HC(C)H-TOCSY, HCC(CO)NH, and (H)CCH- TOCSY spectra were acquired. Aromatic atoms were assigned by the help of 2D CB(CGCD)HD and CB(CGCDCE)HE spectra. The NOE distance restraints were collected from 2D ^{1}H-^{1}H NOESY as well as ^{15}N- and ^{13}C-resolved 3D NOESY experiments. The disulfide pattern of $NFAP_2$ was determined in a collaborative study [2] and the bonds were added as covalent bond restraints for the structure calculation. Backbone torsional angle restraints (determined by TALOS-N webserver) were also included in the process. The structure calculation carried out in two steps. The initial fold of $NFAP_2$ was determined using only NOE distance restraints. In the second, structure refinement step disulfide bonds and the 68 backbone torsional angle restraints were also included. In the final cycle 1102 NOEs were considered. Overall ensembles of 100 structures were calculated and the 20 of them with the lowest energy were chosen for the final structure model.
Finally, the following software packages were used for the structure determination of NFAP2: Topspin 3.1 (spectra processing), CCPNmr Analysis 2.5.2 (chemical shift assignment and peak picking) and Unio10/Cyana 2.1 (structure calculation and refinement).
